# Poxvirus H5 mediates the formation of liquid-liquid phase separation condensates which promote virus factory assembly

**DOI:** 10.1371/journal.ppat.1013708

**Published:** 2025-11-20

**Authors:** Junda Zhu, Zihui Zhang, Yongxiang Fang, Jian Xu, Zhimin Jiang, Hua Li, Shijie Xie, Kang Niu, Zhizhong Jing, Baifen Song, Wenxue Wu, Chen Peng

**Affiliations:** 1 National Key Laboratory of Veterinary Public Health, College of Veterinary Medicine, China Agricultural University, Beijing, China; 2 Lanzhou Veterinary Research Institute, Chinese Academy of Agricultural Sciences, Lanzhou, China; Pirbright Institute, UNITED KINGDOM OF GREAT BRITAIN AND NORTHERN IRELAND

## Abstract

Liquid-liquid phase separation (LLPS) is a fundamental mechanism for the formation of membrane-less organelles, enabling cells to compartmentalize biochemical processes without membrane boundaries. In viral infections, LLPS is increasingly recognized as a strategy for organizing replication and transcriptional machinery. Here, we report that H5, a DNA-binding protein of vaccinia virus (VACV) could undergo LLPS through its N-terminal intrinsically disordered region (IDR). H5 forms dynamic and reversible condensates in both transfected and vacv infected cells, a property also observed with H5 orthologs from mpox virus and lumpy skin disease virus. Fluorescence recovery after photobleaching (FRAP) assays confirmed the liquid-like behavior of H5 condensates. Using structure-guided mutagenesis and phosphoproteomics, we identified two critical phosphorylation sites within the IDR, S127 and S130, which are essential for the interaction between H5 and DNA. These modifications are mediated redundantly by host proteins and viral B1 kinases. Mutations at these residues inhibit the binding of H5 to DNA, thereby directly or indirectly abolish LLPS formation, and impair viral replication factory assembly, leading to a marked reduction in viral DNA replication and progeny production, without affecting the synthesis of H5 or its subcellular localization. Our findings indicate that these two serine residues of H5 contribute to its interaction with DNA and the formation of LLPS, a process that may help organize viral replication compartments and facilitate interactions with key components of the DNA polymerase complex. This study uncovers a previously uncharacterized mechanism by which the poxvirus H5 protein promotes viral factory assembly and coordinates replication, and identifies a conserved regulatory axis that may serve as a potential therapeutic target across poxvirus species.

## Author summary

In this study, we investigate the role of a protein called H5 from the vaccinia virus in the formation of structures inside infected cells known as viral factories. These viral factories are essential for the virus to replicate efficiently. We found that a specific region of the H5 protein enables it to form condensates with LLPS properties. These dense, liquid-like clusters may facilitate the organization of viral components and are critical for viral replication. By altering specific amino acids in H5, we were able to disrupt its ability to form these condensates, which severely affected the virus’s ability to replicate without changing how the protein is made or where it is located in the cell. Our research shows that phosphorylation of H5, a chemical modification, is crucial for its ability to form these condensates and support viral replication. This work opens new avenues for understanding how viruses manipulate cellular processes and provides potential targets for developing antiviral therapies aimed at preventing viral replication by interfering with H5–DNA binding and H5-mediated LLPS.

## Introduction

Liquid-liquid phase separation (LLPS) is the process by which a homogeneous liquid spontaneously demixes into two distinct liquid phases [1–4]. Biopolymers capable of mediating LLPS can transiently interact to form dense, membraneless condensates. These condensates play a crucial role in organizing various biological processes [5–12]. Proteins that facilitate the formation of condensates often exhibit key features, such as intrinsically disordered regions (IDRs) and the ability to interact with nucleic acids [1,3,8]. IDRs provide structural flexibility, a vital component for LLPS, and are frequently enriched in sites prone to post-translational modifications (PTMs), such as phosphorylation [13–17]. By driving the formation of these dynamic and membraneless compartments, LLPS enables cells to efficiently modulate biochemical reactions, respond to environmental changes, and maintain homeostasis.

Recent reports highlight that some viruses have evolved the ability to exploit LLPS to form condensates, establishing specialized compartments within host cells to concentrate viral components or evade immune surveillance [18]. For example, Epstein-Barr virus proteins EBNA2 and EBNALP form condensates within the nucleus of infected cells to promote viral gene transcription [19]. Similarly, the human cytomegalovirus (HCMV) protein UL112–113 forms condensates to establish replication compartments in the nucleus, a process essential for recruiting the viral DNA polymerase [20]. The nucleocapsid protein of SARS-CoV-2 also enhances viral replication by forming condensates that disrupt the cGAS-STING complex and facilitate viral genome packaging [21–23]. By exploiting LLPS, viruses can efficiently hijack host cellular mechanisms to create an optimized microenvironment for their replication. A deeper understanding of these regulatory mechanisms may provide potential targets for antiviral therapies.

Poxviruses, including mpox virus (MPXV) and vaccinia virus (VACV), are large double-stranded DNA viruses that replicate exclusively in the cytoplasm of infected cells [24,25]. Upon entry, discrete cytoplasmic viral factories (VFs) assemble at perinuclear sites to support viral transcription and DNA replication; in VACV-infected cells, these factories are tightly wrapped by endoplasmic reticulum–derived membranes, forming “mini-nuclei” that promote efficient replication [24–26]. VACV H5 (encoded by H5R) is an abundant early gene product that is also expressed later in infection, originally purified as a component of the post-replicative (late) transcription machinery (VLTF-4) and found on nascent viral DNA together with replisome proteins [27,28]. Biochemical analyses show that H5 preferentially binds double-stranded nucleic acids in a sequence-independent manner and forms higher-order oligomers, consistent with a scaffold-like role in organizing replication and transcription modules [29]. Genetic studies underscore H5’s multifunctionality: a dominant temperature-sensitive mutant exhibits profound defects in virion morphogenesis, while deletion of H5 abrogates viral DNA synthesis, confirming its essential role in replication and morphogenesis [30,31]. In addition, H5 is also a phosphoprotein that interacts with the viral B1 kinase and is phosphorylated in this context; hypophosphorylated H5 preferentially localizes to virosomes, and loss of B1 can be partially complemented by the cellular VRK2 kinase, indicating regulation by host–virus kinases [32,33]. In addition, H5 associates with viral mRNA processing and post-replicative transcription machinery, further highlighting its multifunctional role [34,35]. Emerging evidence suggests that poxvirus factories behave as biomolecular condensates formed via LLPS, with H5 nominated as a key scaffold or driver that organizes factory assembly and maturation, providing a mechanistic framework for how its nucleic-acid binding, oligomerization, and protein interactions coordinate the spatial and functional organization of viral replication compartments [29,36]. Collectively, these data portray H5 as a multifunctional hub bridging nucleic-acid binding, transcription, DNA replication, and virion morphogenesis.

Through screening for VACV proteins with evident IDRs, we identified H5 as a candidate, as it contains a 134 amino acid IDR at the N-terminus. Confocal microscopy analysis of transfected or virally expressed H5 indicated that H5 formed puncta within the cytoplasm of infected cells. Fluorescence recovery after photobleaching (FRAP) assay confirmed that the condensates formed by H5 exhibited properties consistent with LLPS. These phenomena are not limited to VACV as H5 orthologs form lumpy skin disease virus (LSDV) and MPXV also form condensates upon transfection. Through alanine substitution and mass spectrometry analyses, we identified specific phosphorylated residues on H5 that are critical for its oligomerization, DNA binding, and condensates formation, which in turn are essential for the assembly of viral replication factories and successful viral replication. Our findings provide deeper insights into the mechanism by which the poxvirus H5 protein promotes viral factory assembly, enhance our understanding of the multifunctional role of H5 in the poxvirus life cycle, and offer potential targets for antiviral therapy.

## Results

### H5 mediates the formation of LLPS condensates in poxviruses

**Fig 1:**
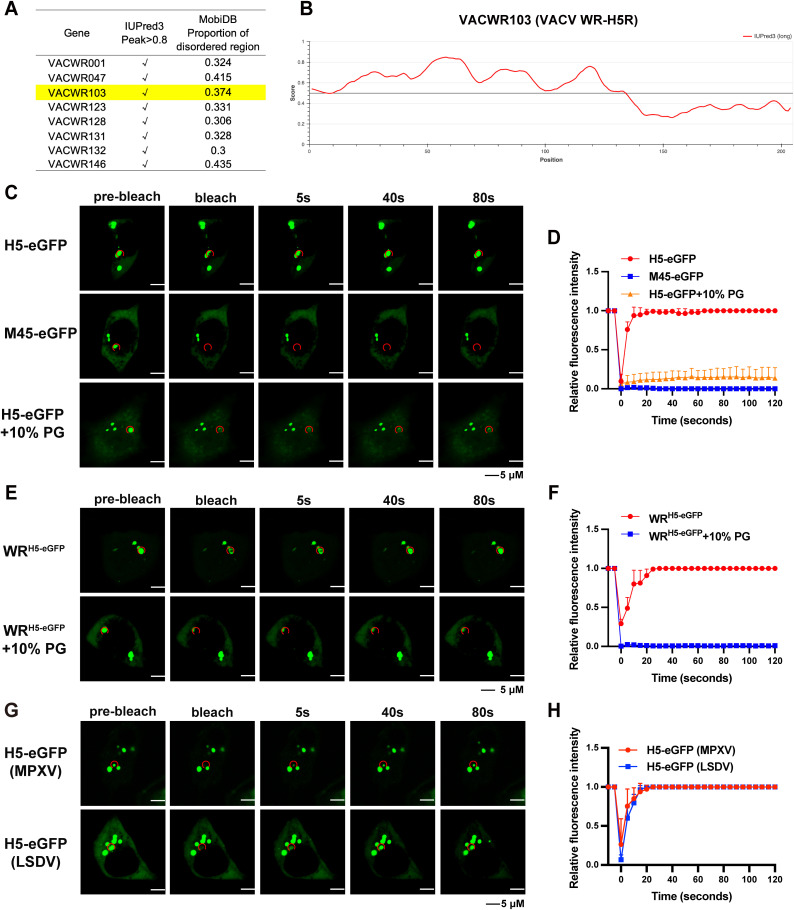
H5 forms LLPS condensates formation under transfection or infection conditions. (A) All viral proteins of VACV-WR were predicted by IUPred3 and MobiDB. All 8 proteins with IUPred3 disorder degree peak value higher than 0.8 and MobiDB predicted disordered region ratio greater than 0.3. (B) Prediction results of H5 protein on IUPred3. The score represents the degree of protein disorder, and regions higher than 0.5 are considered intrinsically disordered regions. (C and D) A549 cells were transfected with the H5-eGFP or M45-eGFP for 24 hours. H5-transfected cells were treated with 10% PG (v/v) for 1 hour. Fluorescence recovery was analyzed by FRAP (C), and relative fluorescence intensity versus time was recorded (D). Scale bars are shown at bottom. (E and F) A549 cells were infected with the WR^H5-eGFP^ at 3 PFU/cell for 4 hours. WR^H5-eGFP^-infected cells were treated with 10% PG (v/v) for 1 hour. Fluorescence recovery was analyzed by FRAP (E), and relative fluorescence intensity versus time was recorded (F). Scale bars are shown at bottom. (G and H) A549 cells were transfected with the H5-eGFP (MPXV) or H5-eGFP (LSDV) for 24 hours. Fluorescence recovery was analyzed by FRAP (G), and relative fluorescence intensity versus time was recorded (H). Scale bars are shown at bottom. Data are mean ±SD (standard deviation). n = 3.

Since proteins that mediate LLPS condensates formation often contain an IDR, we analyzed all protein-coding sequences in the VACV-WR (Western Reserve strain) genome using IUPred3 and MobiDB [37,38]. A total of eight viral proteins exhibited a disordered degree higher than 0.8 in IUPred3 analysis and higher than 0.3 in MobiDB analysis (Fig 1A). Among them, VACV H5 (VACV-WR103) is the only one that contains a nucleic acid binding region, which is often found in other viral proteins that can promote the formation of condensates. Next, we constructed a chimeric protein by fusing the coding sequence of an enhanced green fluorescent protein (eGFP) to the C-terminus of H5, as the disordered region (Score > 0.5) was predominantly located in the N-terminus (1–134, Fig 1B). To detect whether the morphology of H5 during transfection is consistent with infection and the effect of eGFP fusion on its morphology, we first used the purified H5 protein to immunize rabbits and prepared a monoclonal antibody against VACV H5. Western blotting analysis confirmed that the antibody could specifically recognize H5 (S1A Fig). Subsequently, the antibody was employed to detect the morphology of H5 under different infection or transfection conditions (S1B Fig). The results showed that H5 formed small round or oval puncta at 2 hours post-infection (hpi). Transfected H5 exhibited a similar morphology, also appearing as dense puncta, although the number of puncta was lower compared with infection. The presence of an eGFP tag did not noticeably alter this morphology. Thus, transfected H5 can partially recapitulate the puncta morphology observed during VACV infection. In addition, unless otherwise specified, H5 and its truncations or mutants used in subsequent experiments all expressed an eGFP tag at the C-terminus. A negative control construct was generated by fusing eGFP to the C-terminus of murine cytomegalovirus M45, a protein known to form puncta without displaying any LLPS properties [39,40]. The two constructs were cloned into a mammalian expression vector and were used to transfect human A549 cells plated on coverslips. At 24 hours post-transfection, cells were subjected to a FRAP assay, which is an assay often used to examine LLPS properties of puncta [3,8]. At the same time, 10% 1,2-propylene glycol (PG) (v/v), an aliphatic diol that can interfere with the LLPS properties of puncta, was added to the H5 transfected cells, and FRAP assay was performed after 1 hour of PG treatment [41]. The recovery of fluorescent signals was measured and recorded after photobleaching and relative fluorescent intensity was calculated and plotted (Fig 1C and 1D). Transfection of H5-eGFP and M45-eGFP led to the formation of fluorescent puncta, but only those formed by H5 exhibited rapid recovery after photobleaching (Fig 1C and 1D). The fluorescent signals of H5-eGFP were able to recover to the same level prior to photobleaching within 15 seconds while those of M45 were unable to. The recovery of fluorescent signals of H5-eGFP was inhibited by PG, further demonstrating the LLPS properties of these puncta. It is worth noting that H5 puncta did not disappear after the addition of PG, which may be related to the oligomerization or multimerization characteristics of H5. These results indicate that H5 forms condensates rather than simple protein puncta.

To verify whether H5 can also form condensates during viral infection, a recombinant VACV was generated by replacing the ORF of H5 in VACV-WR with an H5-eGFP cassette without disturbing its natural viral promoter (WR^H5-eGFP^). The modification to H5 ORF did not alter viral replication kinetics as the recombinant virus WR^H5-eGFP^ was able to replicate to comparable titers as VACV-WR (S2 Fig). A549 cells were then infected with WR^H5-eGFP^ and a FRAP assay was performed at 4 hpi as described above. Similar to the results observed in cells transiently transfected with H5-eGFP, fluorescent puncta were observed at 4 hpi and the signals were able to recover after photobleaching within 15 seconds (Fig 1E and 1F). Whether the addition of 10% PG during WR infection affects the recovery of H5 fluorescence was also verified. Similar to transfected H5, PG did not induce the disappearance of H5 puncta during WR^H5-eGFP^ infection, but the puncta no longer displayed LLPS properties.

To determine if the ability to form condensates is conserved in other poxviruses, we first made an alignment of H5 by including thirteen H5 sequences from various poxviruses. This analysis indicated that the N-terminal IDR is relatively more conserved across different poxviruses despite single nucleotide variations found (S3 Fig). We selected MPXV, a member of the *Orthopoxvirus*, and LSDV, a member of the *Capripoxviruses*, and cloned their H5 sequences into the same mammalian expression vector that contains eGFP. A549 cells were transfected with MPXV-H5-eGFP or LSDV-H5-eGFP, and FRAP analyses were performed as described above. Similar to VACV-WR, fluorescent puncta that were able to recover rapidly from photobleaching were observed in cells transfected with MPXV-H5-eGFP or LSDV-H5-eGFP (Fig 1G and 1H), indicating the capability of forming LLPS condensates was not specific for VACV H5 but conserved in other poxviruses.

### The intrinsically disordered region of H5 dictates its LLPS properties

**Fig 2:**
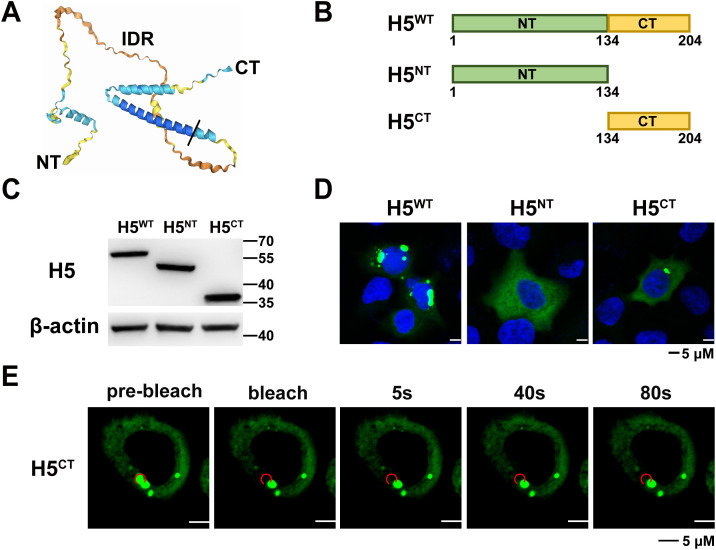
The CT enables H5 to maintain puncta, while the IDR endows H5 with LLPS properties. (A) Protein structure prediction of H5. IDR, intrinsically disordered region; NT, N-terminal; CT, C-terminal. (B) Schematic diagram of H5 protein truncation. (C) A549 cells were transfected with the full-length H5^WT,^ H5^NT^, or H5^CT.^ After 24 hours, cells were lysed and detected by Western blotting using anti-H5 and anti-β-actin antibodies. (D) A549 cells were transfected with the full-length H5^WT^, H5^NT^, or H5^CT^. After 24 hours, cells were then fixed, permeabilized, blocked, and Hoechst was used to stain DNA. Scale bars are shown at bottom. (E) A549 cells were transfected with the H5^CT^ for 24 hours. Fluorescence recovery was analyzed by FRAP. Data are mean ±SD. n = 3.

To map the functional domains of H5 required for condensates formation, AlphaFold3 was employed to predict the structure of VACV H5 [42,43]. The predicted structure presented a relatively clear boundary between the CT (C-terminal) and IDR (Fig 2A). Based on the simulated structure and the analysis from IUPred3, the amino acid sequence of H5 was divided into two domains, namely NT (N-terminal, a.a.1-134), which is almost entirely IDR, and CT (a.a.135-204), which contains a long α-helix. Two truncated mutants were generated retaining only NT or CT (Fig 2B). Both mutants were then fused with eGFP and the successful synthesis of the recombinant proteins was examined by Western blotting analysis using an antibody for eGFP (Fig 2C). A549 cells were transfected with the full-length H5^WT^ or the two truncated mutants, and confocal microscopic analysis was performed. While the CT expressed alone could form puncta, the IDR transfected alone exhibited a diffuse pattern within the cytoplasm and was no longer able to form puncta (Fig 2D). To further examine the puncta observed in H5^CT^-transfected cells, a FRAP analysis was performed (Fig 2E). Unlike full-length H5^WT^, fluorescent signals from the H5^CT^ were not able to recover after photobleaching, indicating that puncta formed by the CT of H5 alone may resemble those observed after PG treatment, retaining a punctate morphology without dynamic recovery. These data demonstrated that the CT is essential for the formation of H5 puncta, while the IDR contributed to its LLPS properties.

### The S127 and S130 sites in the IDR are the key phosphorylation sites for H5-mediated LLPS

**Fig 3:**
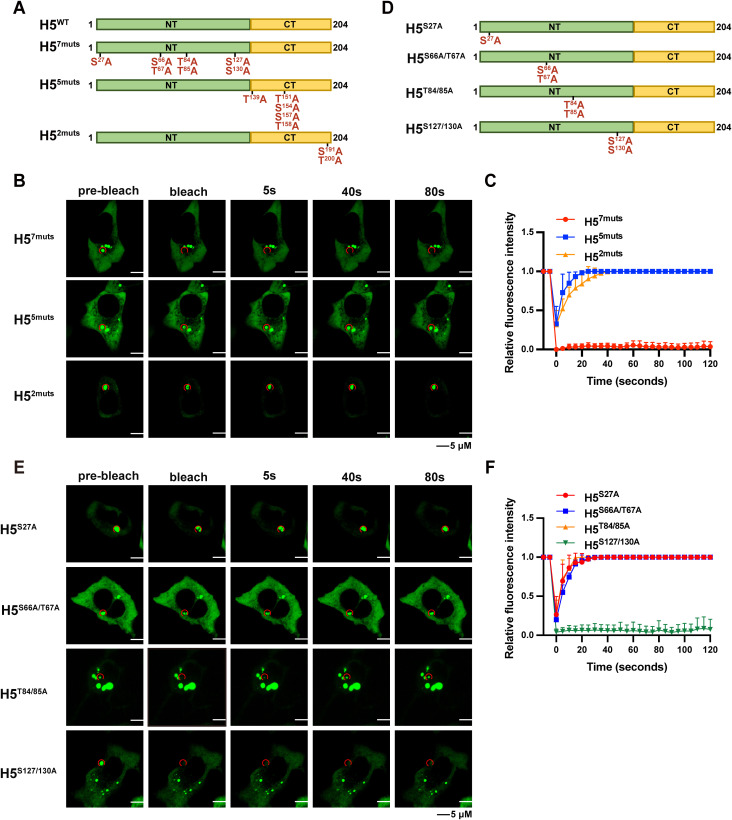
Screening of key phosphorylation sites of H5 condensate formation. (A) Schematic diagram of H5 combinatorial mutants. (B and C) A549 cells were transfected with the H5^7muts,^ H5^5muts^, or H5^2muts^ for 24 hours. Fluorescence recovery was analyzed by FRAP (B), and relative fluorescence intensity versus time was recorded (C). (D) Schematic diagram of H5 mutants. (E and F) A549 cells were transfected with the H5^S27A^, H5^S66A/T67A^, H5^T84/85A^, or H5^S127/130A^ for 24 hours. Fluorescence recovery was analyzed by FRAP (E), and relative fluorescence intensity versus time was recorded (F). Data are mean ±SD. n = 3.

Previous studies published by Kay et al. indicated that phosphorylation of H5 modulates its multimerization [29]. To examine whether phosphorylation of H5 drives the formation of condensates by promoting its multimerization, all serine and threonine residues were mutated to alanine in different combinations (Fig 3A). Three H5 mutants were constructed, including H5-7mutant (H5^7muts^) with 7 mutations in the NT, and H5-5mutant (H5^5muts^) with 5 mutations or H5-2 mutant (H5^2muts^) with 2 mutations in the CT (Fig 3A). A549 cells were transfected with the above mutants and the recovery of fluorescent signals was captured by the FRAP assay. None of the introduced mutations impaired the ability of H5 to form puncta within cells. However, 7 mutations in the NT caused H5 puncta to lose its LLPS properties and were no longer able to recover after photobleaching (Fig 3B and 3C), suggesting phosphorylation of one or multiple residues within this region was essential for H5 to form condensates. Next, these seven residues were further divided into four groups, resulting in the generation of four additional constructs H5^S27A^, H5^S66A/T67A^, H5^T84/85A^, and H5^S127/130A^ (Fig 3D). The FRAP assay was conducted using these mutants and only H5^S127/130A^ was no longer able to form puncta with LLPS properties, these data indicated that the two serine residues in the IDR were crucial for H5 condensates formation (Fig 3E and 3F).

**Fig 4:**
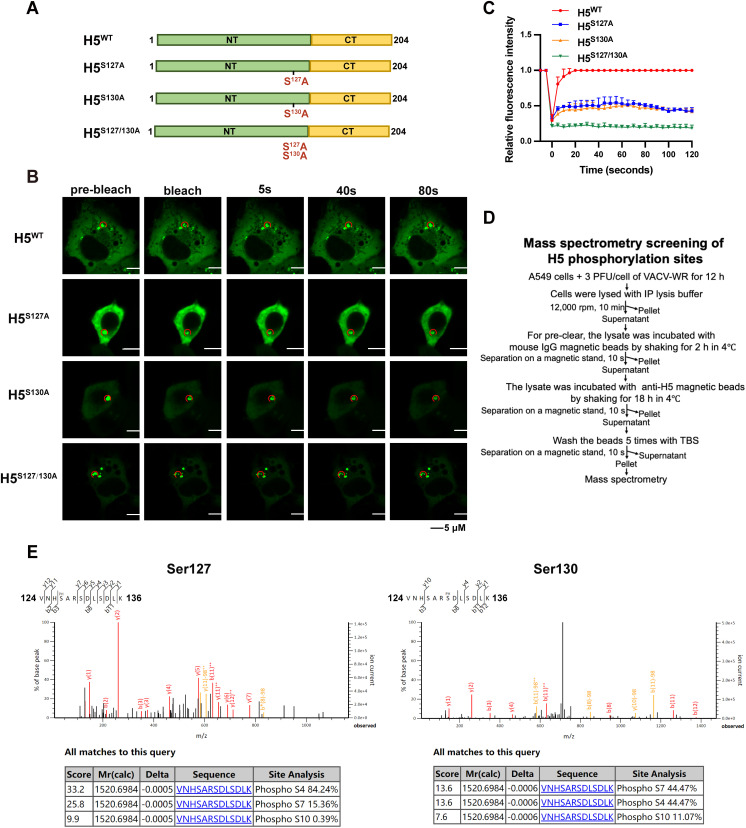
The potential phosphorylation sites S127 and S130 are critical for H5 condensate formation. (A) Schematic diagram of H5 mutants. (B and C) A549 cells were transfected with the H5^WT^, H5^S127A^, H5^S130A^, or H5^S127/130A^ for 24 hours. Fluorescence recovery was analyzed by FRAP (B), and relative fluorescence intensity versus time was recorded (C). (D) Schematic flow chart of mass spectrometric screening of H5 phosphorylation sites. (E) MS^2^ spectrum of the tyrosine-phosphorylated peptide from H5. The pronounced neutral loss of phosphoric acid (−98) marked with * indicated serine as the modification site. The fragment ions whose m/z value corresponds to y or b ions with the loss of a phosphate group are indicated. The MS^2^ spectrum indicated that Ser127 and Ser130 from H5 are highly likely phosphorylation modification sites. Data are mean ±SD. n = 3.

Single nucleotide mutations were then made and the impact of these residues on the LLPS properties of H5 puncta was examined by the FRAP assay (Fig 4A). The mutation of only S127 or S130 reduced the fluorescence recovery of H5 puncta, demonstrating that S127 and S130, two residues conserved among poxviruses (S3 Fig), synergistically contributed to the formation of condensates (Fig 4B and 4C). As we lack antibodies that were able to recognize only phosphorylated H5, to verify these residues mutated were indeed phosphorylated during VACV infection, a mass-spectrometry analysis was performed (Fig 4D). A549 cells were infected with VACV-WR. Cell lysates were then collected and a co-immunoprecipitation (co-IP) assay was conducted by pulling down H5 with the monoclonal antibody generated in the lab. Mass-spectrometry was employed to determine the phosphorylated residues on H5 (Fig 4D and 4E). According to the Mascot Delta Score, by analyzing the MS^2^ spectrum of the serine-phosphorylated peptide from H5, it was found that both Ser127 and Ser130 from H5 were phosphorylation modification sites [44]. These results indicated that S127 and S130 were indeed phosphorylated during VACV infection.

### Host protein VRK2 is involved in H5 phosphorylation and condensates formation

**Fig 5:**
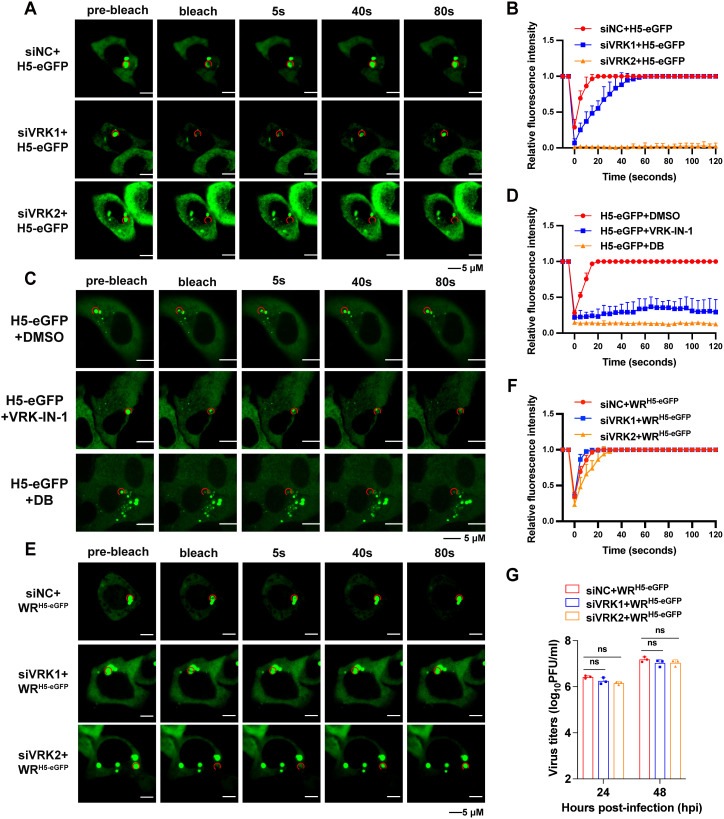
VRK2 is essential for H5 condensate formation under transfection conditions. (A and B) A549 cells were transfected with the siNC, siVRK1-1, or siVRK2-2 for 48 hours, then transfected with H5 for 24 hours. Fluorescence recovery was analyzed by FRAP (A), and relative fluorescence intensity versus time was recorded (B). (C and D) A549 cells were treated with the inhibitors of VRK1 (VRK-IN-1, 20 μM) or VRK2 (DB, 20 μM) for 48 hours, then transfected with H5 for 24 hours. Fluorescence recovery was analyzed by FRAP (C), and relative fluorescence intensity versus time was recorded (D). (E and F) A549 cells were transfected with the siNC, siVRK1-1, or siVRK2-2 for 48 hours, then infected with WR^H5-eGFP^ at 3 PFU/cell for 4 hours. Fluorescence recovery was analyzed by FRAP (E), and relative fluorescence intensity versus time was recorded (F). (G) A549 cells were transfected with siNC, siVRK1 or siVRK2 and then infected with WR^H5-eGFP^ at 0.01 PFU/cell. Viruses were harvested at 24 or 48 hpi and quantified by plaque assay. Data are mean ±SD. n = 3. (G) two-sided Student’s t test; ns, not significant.

Previous studies have reported that both the viral B1 kinase and the host vaccinia-related kinases (VRKs) participate in phosphorylation pathways during VACV infection and have been identified as potential kinases responsible for phosphorylating H5 [45–49]. In addition, deletion of the viral B1 kinase has been shown to reveal its essential functions, which can be partially compensated by the homologous host kinase VRK2 [32]. To determine if cellular kinases VRK1 and VRK2 were responsible for H5 phosphorylation and the subsequent formation of condensates, the knockdown efficiency of VRK1/VRK2 by different siRNAs was first tested (S4A and S4B Fig). Cellular VRK1 or VRK2 were knocked down with siRNA and the cells were then transfected with H5-eGFP (Fig 5A and 5B). Although suppression of VRK1 slightly delayed the recovery of fluorescent signals, the suppression of VRK2 completely abolished the signal recovery, demonstrating its fundamental role in promoting the formation of fluorescent condensates (Fig 5A and 5B). Pharmaceutical inhibitors of VRK1 (VRK-IN-1, 20µM) and VRK2 (Dihydrobaicalein, DB, 20µM) were next used to assess the role of these two VRKs on the formation of condensates. Consistent with siRNA knockdown, the VRK-2 inhibitor dihydrobaicalin completely abolished the ability of transfected H5 to form condensates, while the VRK-1 inhibitor VRK-IN-1 also exhibited a partial effect, which may be due to off-target effects (Fig 5C and 5D).

Furthermore, as previous studies suggested that H5 might also be phosphorylated by MAPK family kinases, the effect of MAPK knockdown on H5 condensate formation was examined [47]. The knockdown efficiencies of MAPK3/14/15 were first validated (S5A–C Fig), followed by FRAP analysis to evaluate fluorescence recovery of H5 condensates after knockdown of MAPK3/14/15 (S5D and S5E Fig). Knockdown of MAPK3/14 had no significant impact on fluorescence recovery, whereas MAPK15 knockdown moderately reduced recovery, although to a lesser extent than VRK2 knockdown, suggesting that H5, as an essential gene of VACV, may exploit multiple cellular kinases for its function.

Next, siVRK1/2 knockdown efficiency was assessed under infection conditions and was found to be effective (S4C and S4D Fig). siRNA-mediated inhibition of VRK1/2 was then performed to evaluate their role in H5 phosphorylation and condensate formation during VACV infection (Fig 5E and 5F). The results showed that inhibition of VRK1/2 did not impair the formation of H5 condensates. The viral titers after VRK1/2 knockdown were also tested and there was no significant change (Fig 5G). This may be due to the ability of the viral B1 protein to phosphorylate H5. As B1 is an essential gene, and the deletion of B1 from VACV was unsuccessful, we were unable to test the direct effect of B1 on H5 phosphorylation. These data indicated that viral kinases may compensate for the loss of host kinase VRK2. Overall, our data demonstrated that the phosphorylation of S127 and S130 on H5 was crucial for H5’s ability to form condensates.

### H5 condensates formation requires additional cellular components

**Fig 6:**
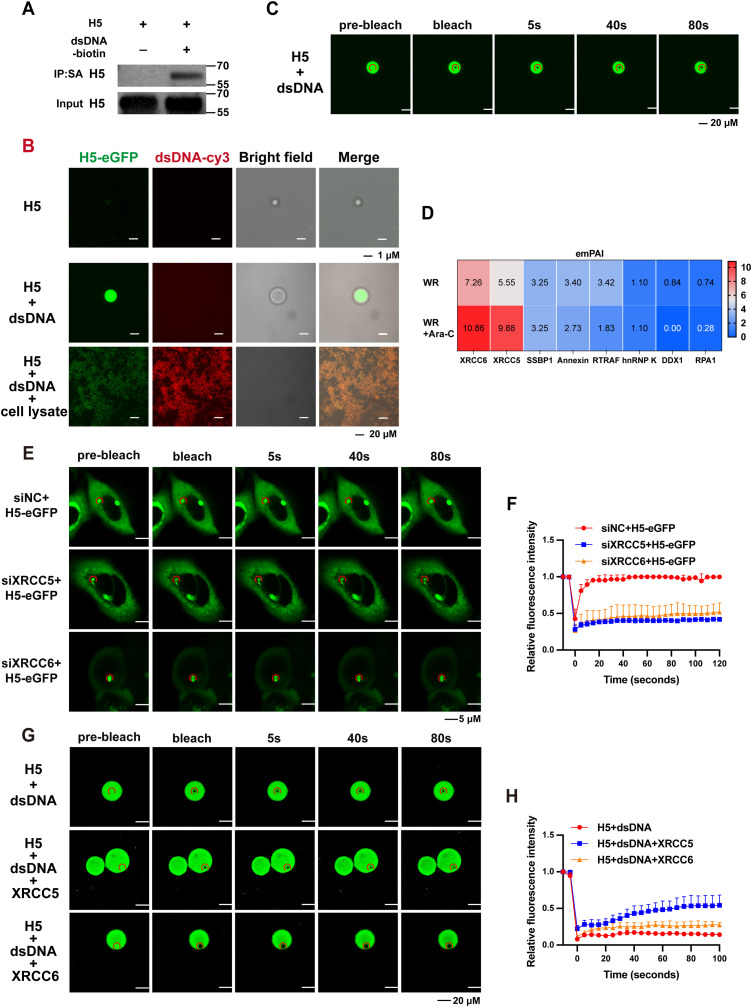
Purified H5 requires DNA and intracellular components to form condensates. (A) The purified H5 protein was incubated with biotin-labeled dsDNA at 4 °C for 18 hours, followed by a 6-hour incubation with streptavidin-coated beads. Proteins were eluted using SDS sample buffer and subsequently analyzed by SDS-PAGE and Western blotting. (B) Purified H5-eGFP alone or in combination with Cy3-labeled dsDNA and cell lysate was incubated in buffer (50 mM Tris-HCl pH 7.4, 175 mM NaCl, 2 mM MgCl_2_, 1 mM ATP, 0.5 mM TCEP, 2.5% PEG 8000) at 25 °C to induce phase separation. Images were acquired using confocal microscope. (C) Purified H5-eGFP was mixed with Cy3-labeled dsDNA at 25 °C to induce phase separation. Fluorescence recovery was analyzed by FRAP. (D) Mass spectrometry was used to identify the host proteins that interacted with H5. The relative content of host proteins was expressed by the exponentially modified protein abundance index (emPAI). (E and F) A549 cells were transfected with the siNC, siXRCC5, or siXRCC6 for 48 hours, then transfected with H5 for 24 hours. Fluorescence recovery was analyzed by FRAP (E), and relative fluorescence intensity versus time was recorded (F). (G and H) Purified H5-eGFP was mixed with Cy3-labeled dsDNA and XRCC5/6 at 25 °C to induce phase separation. Fluorescence recovery was analyzed by FRAP (G), and relative fluorescence intensity versus time was recorded (H). Data are mean ±SD. n = 3. (F and H) two-sided Student’s t test; ns, not significant.

To further characterize the phase separation properties of H5, we established an H5-expressing stable cell line in which the expression of His-tagged H5 was inducible by doxycycline (Dox) and the cell line was named A549^H5-eGFP^. The clones were selected based on H5 expression and the synthesis of H5 was confirmed visa Western blotting analysis (S6 Fig). H5 was purified using its Ni-column and subsequently evaluated for its ability to directly bind double-stranded DNA (dsDNA) in the absence of cellular cofactors. Biotin-labeled interferon-stimulatory DNA (ISD) was synthesized and incubated overnight at 4°C with purified H5 protein, followed by incubation with streptavidin beads [50]. The results demonstrated that H5 directly binds dsDNA in cell-free system (Fig 6A). Further examination revealed that purified H5 alone, in the absence of dsDNA, exhibited a diffuse distribution (Fig 6B). Co-incubation of H5 with dsDNA resulted in the formation of punctate resembling those observed in cells (Fig 6B); however, fluorescence recovery was not detected after FRAP analysis, indicating that these puncta do not exhibit LLPS properties (Fig 6C). These findings suggest that additional cellular cofactors may be required for LLPS. When cell lysate was added to the H5-dsDNA mixture, H5 formed amyloid-like solid phase separation (LSPS) structures (Fig 6B), resembling that formed by cGAS in a cell-free system [51]. Collectively, these results indicate that H5 possesses phase separation potential, predominantly forming LSPS in purified conditions, whereas LLPS may occur in cells in the presence of partner proteins.

To identify host proteins that may cooperate with H5 in condensate formation, we applied the same mass spectrometry strategy used in Fig 4, this time including Cytarabine (Ara-C) to minimize interference from DNA replication. Through this approach, cellular proteins interacting with H5 were identified (Fig 6D), among which XRCC family members XRCC5 and XRCC6 emerged as the most enriched interactors. Knockdown of XRCC5/6 in A549 cells markedly reduced the fluorescence recovery of H5 condensates in FRAP assays (Fig 6E and 6F). Furthermore, His-tagged XRCC5/6 constructs were generated, and the proteins were expressed and purified from HEK-293T cells. When purified XRCC5 or XRCC6 was added to the H5–dsDNA system, fluorescence recovery of the condensates increased compared to the H5–dsDNA control, with XRCC5 exhibiting a more pronounced effect than XRCC6. However, neither protein restored fluorescence to pre-bleach levels (Fig 6G and 6H). FRAP analyses were performed on three droplets in the same experiment, with results shown in Fig 6G and S7 Fig. These results suggest that both XRCC5 and XRCC6 facilitate H5-mediated LLPS, but additional cellular or viral factors are likely required for full condensate fluorescent recovery.

### H5 multimerization and DNA binding promote LLPS and viral replication

**Fig 7:**
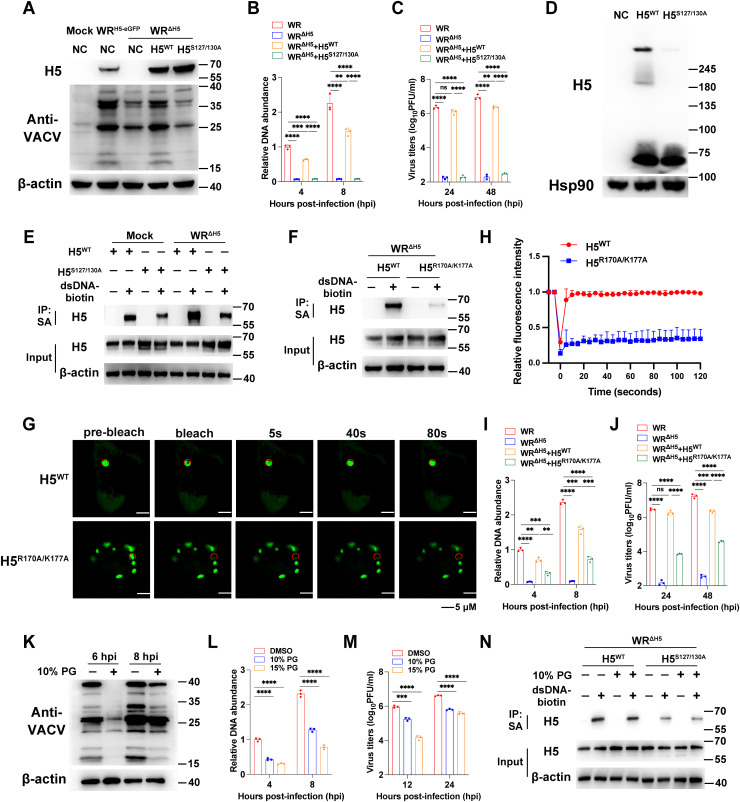
Disruption of H5 condensates significantly inhibits VACV replication. (A) A549 cells were transfected with H5^WT^ or H5^S127/130A^ and then infected with WR^H5-eGFP^ or WR^ΔH5^ at 3 PFU/cell. After 4 hours, cells were lysed and cellular proteins were analyzed by Western Blotting. (B) A549 cells were transfected with H5^WT^ or H5^S127/130A^ and then infected with WR or WR^ΔH5^ at 3 PFU/cell. Total DNA was harvested at 4 or 8 hpi and viral genomic DNA levels were determined by RT-qPCR. (C) A549 cells were transfected with H5^WT^ or H5^S127/130A^ and then infected with WR or WR^ΔH5^ at 0.01 PFU/cell. Viruses were harvested at 24 or 48 hpi and quantified by plaque assay. (D) A549 cells were transfected with H5^WT^ or H5^S127/130A^ for 24 hours. Cells were lysed using a non-reducing lysis buffer, and the cell lysate was processed and detected by Western Blotting under non-reducing conditions. (E) A549 cells were transfected with H5^WT^ or H5^S127/130A^ for 24 hours and infected with WR^ΔH5^ at 3 PFU/cell for 6 hours. Cell lysates were incubated with streptavidin-trap beads without dsDNA-biotin, or dsDNA-biotin-streptavidin-trap beads at 4˚C for 18 hours. Proteins were eluted with SDS-loading buffer and analyzed by SDS-PAGE and Western blotting. (F) A549 cells were transfected with H5^WT^ or H5^K170A/R177A^ for 24 hours and infected with WR^ΔH5^ at 3 PFU/cell for 6 hours. Cell lysates were incubated with streptavidin-trap beads without dsDNA-biotin, or dsDNA-biotin-streptavidin-trap beads at 4˚C for 18 hours. Proteins were eluted with SDS-loading buffer and analyzed by SDS-PAGE and Western blotting. (G and H) A549 cells were transfected with the H5^WT^ or H5^K170A/R177A^ for 24 hours. Fluorescence recovery was analyzed by FRAP (G), and relative fluorescence intensity versus time was recorded (H). (I) A549 cells were transfected with H5^WT^ or H5^K170A/R177A^ and then infected with WR or WR^ΔH5^ at 3 PFU/cell. Total DNA was harvested at 4 or 8 hpi and viral genomic DNA levels were determined by RT-qPCR. (J) A549 cells were transfected with H5^WT^ or H5^K170A/R177A^ and then infected with WR or WR^ΔH5^ at 0.01 PFU/cell. Viruses were harvested at 24 or 48 hpi and quantified by plaque assay. (K) A549 cells were infected with WR at 3 PFU/cell. WR-infected cells were treated with 10% PG (v/v) for 1 hour at 4 hpi and then removed. After 6 or 8 hours, cells were lysed and cellular proteins were analyzed by Western Blotting. (L) A549 cells were infected with WR at 3 PFU/cell. WR-infected cells were treated with 10% or 15% PG (v/v) for 1 hour at 2 hpi and then removed. Total DNA was harvested at 4 or 8 hpi and viral genomic DNA levels were determined by RT-qPCR. (M) A549 cells were infected with WR at 3 PFU/cell. WR-infected cells were treated with 10% or 15% PG (v/v) for 1 hour at 4 hpi and then removed. Viruses were harvested at 12 or 24 hpi and quantified by plaque assay. (N) A549 cells were transfected with H5^WT^ or H5^S127/130A^ for 24 hours and infected with WR^ΔH5^ at 3 PFU/cell. WR^ΔH5^-infected cells were treated with 10% PG (v/v) for 2 hours at 4 hpi. At 6 hpi, cells were lysed and the lysates were incubated with streptavidin-trap beads without dsDNA-biotin, or dsDNA-biotin-streptavidin-trap beads at 4˚C for 18 hours. Proteins were eluted with SDS-loading buffer and analyzed by SDS-PAGE and Western blotting. Data are mean ±SD. n = 3. (B, C, I, J, L and M) two-sided Student’s t test; **p < 0.01, ***p < 0.001, ****p < 0.0001, ns, not significant.

Since phosphorylation of S127 and S130 is essential for H5 condensates formation, the next step is to determine how mutations at these sites specifically affect H5 function and whether they significantly impact viral replication. To better investigate the function of H5 during viral infection and its impact on viral biological characteristics, we decided to generate an H5-knock-out virus using VACV-WR. Since deletion of H5 is an essential gene for viral replication, the inducible H5-expressing cell line (A549^H5-eGFP^) was used. Under dox induction, H5 expression was activated in these cells, and the H5 ORF was successfully replaced with mCherry to generate the H5 knockout virus, namely WR^ΔH5^. Subsequent analysis of viral protein expression following WR^ΔH5^ infection showed that loss of H5 markedly reduced viral protein synthesis (detected with anti-VACV antiserum), whereas complementation with H5^WT^, but not H5^S127/130A^, restored protein synthesis (Fig 7A). Similarly, the deletion of H5 results in impaired viral DNA replication and a significant reduction in virus titers (Fig 7B and 7C). The ectopic expression of H5^WT^ was able to largely rescue the reduction in viral DNA abundance and virus titers, bringing them to slightly lower levels than those observed in VACV-WR-infected cells. In contrast, the expression of H5^S127/130A^, despite having comparable levels to H5^WT^, failed to rescue these outcomes (Fig 7B and 7C).

H5 protein has been shown to be phosphorylated and capable of multimerization, forming higher-order oligomers (>400 kDa) *in vitro*, and phosphorylated MPXV H5 also exhibits self-association and dsDNA-binding ability, suggesting that phosphorylation-regulated multimerization may be a conserved mechanism in both VACV and MPXV [29,47]. To verify whether the mutation of H5 phosphorylation site caused the weakening of its multimerization, A549 cells were co-transfected with H5^WT^ or H5^S127/130A^ and the protein lysates were collected and analyzed with non-reducing SDS-PAGE and Western blotting analysis (Fig 7D). The results showed that H5^WT^ formed obvious tetramerization and polymerization bands after transfection, while H5^S127/130A^ was significantly weakened, indicating that the phosphorylation of S127 and S130 is essential for the multimerization of H5 and the subsequent formation of condensates. The next question was whether the weakened H5 multimerization caused by the S127/130A mutation would interfere with its interaction with dsDNA. To test this, cells were transfected with H5^WT^ or H5^S127/130A^ for 24 hours and then infected with WR^ΔH5^ for 6 hours (Fig 7E). Cell lysates were incubated with streptavidin-biotin magnetic beads with or without ISD. The beads were then harvested and washed, and the proteins that interacted with ISD were subjected to Western blotting analysis for H5. The results showed that both H5^WT^ and H5^S127A/S130A^ interacted with dsDNA; however, the interaction appeared weaker when the two serine residues were mutated. This observation correlates with the reduced LLPS observed for H5^S127A/S130A^, although a direct causal relationship between phosphorylation at these sites and dsDNA binding remains to be further investigated.

Previous studies on the structure of MPXV H5 reported that the R170A/K177A mutations at the H5-dsDNA binding interface impairs viral DNA synthesis, which may be caused by impaired binding of H5 to dsDNA [52]. To further investigate the underlying cause, an H5^R170A/K177A^ mutant expression vector was constructed, and its binding ability to dsDNA was analyzed by co-IP (Fig 7F). The results demonstrated a significant reduction in the dsDNA-binding capacity of H5^R170A/K177A^. Next, it was further analyzed whether the R170A/K177A mutations leads to the loss of LLPS properties by weakening the binding of H5 to dsDNA (Fig 7G and 7H). Interestingly, similar to H5^S127/130A^, H5^R170A/K177A^ exhibited a significantly reduced fluorescence recovery after photobleaching, indicating that H5 condensates formation indeed requires binding to dsDNA. Meanwhile, the effects of H5^R170A/K177A^ on viral DNA replication and virus titers were also examined using the same methods as described above (Fig 7I and 7J). The results showed that complementation with H5^R170A/K177A^ could not fully compensate for the loss of H5 in the WR^ΔH5^, unlike H5^WT^, which almost completely restored viral replication. Notably, viruses transfected with H5^R170A/K177A^ retained partial replication ability, which may be due to the fact that the R170A/K177A mutations only reduced the interaction with dsDNA without disrupting H5 phosphorylation or multimerization. These results indicate that H5’s ability to bind dsDNA is closely associated with its condensates formation and significantly affects viral replication.

To directly analyze the impact of the loss of LLPS properties in H5 puncta on viral replication, PG was added during viral infection to disrupt H5 condensates formation, followed by the assessment of viral protein expression, viral DNA replication, and viral titers (Fig 7K-7M). The results showed that the addition of PG led to a significant reduction in viral replication. Additionally, whether PG interferes with the binding of H5 to dsDNA was examined by co-IP, and the results demonstrated that PG did not affect the interaction between H5 and dsDNA (Fig 7N), suggesting that PG may inhibit viral replication by broadly altering the physical properties of viral condensates, including H5 condensates. Collectively, these experiments show that mutations at the phosphorylation sites S127 and S130 of H5 inhibit condensate formation by disrupting its oligomerization and interaction with double-stranded DNA, whereas mutations K170 and R177 act by directly weakening H5’s interaction with double-stranded DNA. Additionally, PG may interfere with H5-mediated LLPS by altering the physical properties of the condensates. All of these disruptions lead to markedly reduced viral replication, highlighting the critical role of H5 in the viral life cycle.

### Phosphorylation of H5 is critical for viral replication factory assembly

**Fig 8:**
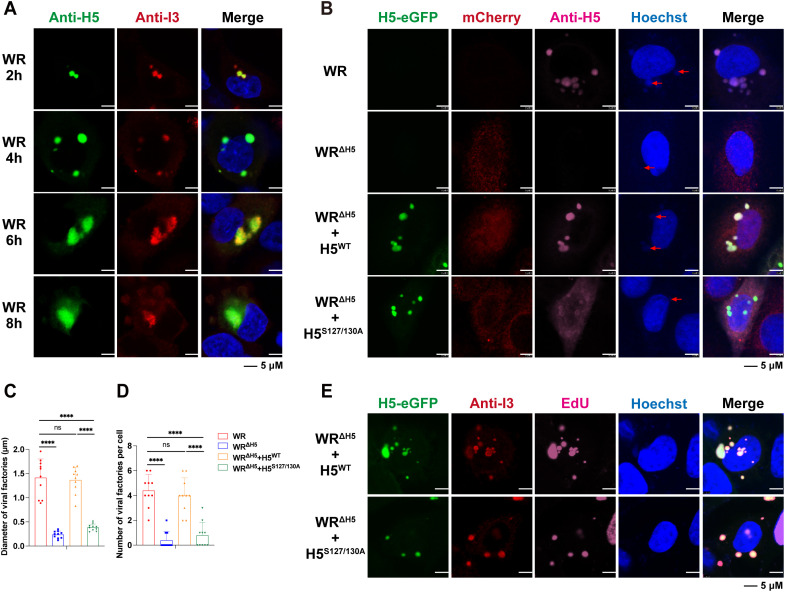
H5 phosphorylation and condensate formation promote viral factory assembly. **(A)** A549 cells were infected with VACV-WR at 3 PFU/cell. After 2, 4, 6 and 8 hours, cells were then fixed, permeabilized, blocked, and stained with primary antibodies to H5 and I3 followed by fluorescent conjugated secondary antibodies. Hoechst was used to stain DNA. Scale bars are shown at bottom. **(B-D)** A549 cells were transfected with H5^WT^ or H5^S127/130A^ and then infected with WR or WR^ΔH5^ at 3 PFU/cell. After 4 hours, cells were then fixed, permeabilized, blocked, and stained with primary antibodies to H5 followed by fluorescent conjugated secondary antibodies. Hoechst was used to stain DNA. Scale bars are shown at bottom **(B)**. Image J was used to count the number of virus factories in 10 randomly selected infected cells **(C)**, and the diameter of 10 randomly selected virus factories **(D)**. **(E)** A549 cells were transfected with H5^WT^ or H5^S127/130A^ and then infected with WR^ΔH5^ at 3 PFU/cell. EdU (10 μM) was added in cells at 2 hpi. The cells were then fixed, permeabilized, blocked, and stained with primary antibodies to H5 and I3 followed by fluorescent conjugated secondary antibodies at 4 hpi. At the same time, a click reaction was performed to connect Azide 647 to EdU. Hoechst was used to stain DNA. Scale bars are shown at bottom. Data are mean ±SD. *n* = 3-10. (C and D) two-sided Student’s t test; ***p < 0.001, ****p < 0.0001, ns, not significant.

Previous studies using temperature-sensitive H5 mutants have shown that the loss of H5 completely blocks viral DNA replication and the assembly of viral replication factories [31]. In addition, it has been reported that B1 and VRK2 can promote the formation of VACV replication factories by inhibiting B12 in a phosphorylation-dependent manner [49]. Given that H5 localizes to viral factories and previous studies have shown that condensates can facilitate the maturation of viral factories or the assembly of replication complexes, it is conceivable that B1 and VRK2 may regulate H5 phase separation through phosphorylation, thereby promoting viral factory maturation [19,20,41]. To investigate this, we first confirmed that H5 localizes to viral factories. A549 cells were infected with VACV-WR at 3 PFU/cell for the indicated time, and viral H5 and I3 were stained with monoclonal antibodies we generated in the lab, or kindly provided by Bernard Moss, respectively. Starting at 2 hpi, viral H5 colocalized with I3 and Hoechst-stained viral factories, and this colocalization persisted until 8 hpi when H5 began to diffuse (Fig 8A). To further investigate the role of H5 phosphorylation and condensates formation in viral factory assembly, A549 cells were transfected with either empty vector, H5^WT^ or H5^S127/130A^ for 24 hours before being infected with WR or WR^ΔH5^ at 3 PFU/cell. Cells were fixed at 4 hpi and analyzed by confocal microscopy. Red arrows indicate the locations of viral factories. Although H5^WT^ largely restored the number of viral factories reduced by H5 depletion, the number of viral factories in cells expressing H5^S127/130A^ was significantly reduced, likely due to weakened H5-DNA binding and impaired condensate formation caused by mutations at the S127 and S130 phosphorylation sites (Fig 8B-D). Moreover, the size of these viral factories was markedly smaller in H5^S127/130A^-transfected cells, further emphasizing the essential role of H5 in the assembly of viral factories (Fig 8D). Further examination of the localization of H5, I3, and nascent DNA in cells transfected with H5^WT^ or H5^S127/130A^ and infected with WR^ΔH5^ was performed. Specifically, during infection, EdU was incorporated into newly synthesized viral DNA, which was subsequently fluorescently labeled via click chemistry to visualize nascent DNA and to mark active viral replication sites (Fig 8E). The results showed that in H5^WT^-transfected cells, H5 colocalized with I3 and nascent DNA within viral factories. In cells transfected with the H5^S127/130A^ mutant, nascent DNA was still detectable and colocalized with H5 and I3, suggesting that viral polymerase activity was not completely inhibited. These findings indicate that loss of H5 phosphorylation at S127 and S130 severely impairs the formation and maturation of viral factories, although viral DNA synthesis and polymerase activity are not completely inhibited. One possible explanation is that loss of phosphorylation impairs H5-mediated LLPS formation. Alternatively, phosphorylation loss may disrupt H5 interactions with other cellular components that are indispensable for viral factory assembly. Collectively, these findings highlight the critical role of H5—particularly its phosphorylation at S127 and S130, and the potential contribution of phosphorylation-driven LLPS—in supporting the proper formation and maturation of viral factories.

## Discussion

**Fig 9:**
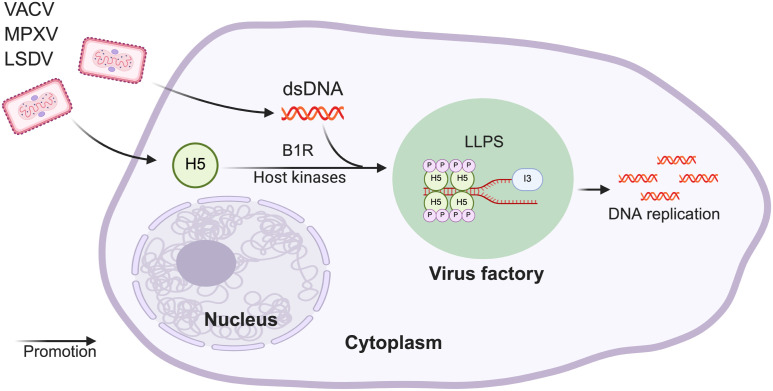
H5 promotes virus factory assembly by mediating LLPS. Upon entry of VACV into host cells, the early protein H5 is expressed. Phosphorylation of H5 at S127 and S130 by the viral kinase B1 or host kinases promotes its oligomerization and binding to nucleic acids, followed by further multimerization to induce LLPS and condensates formation. H5 then cooperates with I3 to facilitate the maturation of viral factories and viral DNA replication. Created in BioRender. Zhu, J. (2025) https://BioRender.com/x5g52m2.

LLPS has been shown to play a crucial role in various biological processes, including nucleolar formation, intracellular signaling, and viral infection and replication [4–12,40]. To explore whether proteins from VACV contribute to condensate formation, we screened and identified H5 as a primary target due to its intrinsically disordered region (IDR) at the N-terminus. Although the IDR did not impact the formation of H5 puncta, it was essential for H5’s LLPS properties, as demonstrated by FRAP analysis. Previous reports indicated that complete removal of H5 severely jeopardized viral replication and virus factory formation, consistent with its essential roles in DNA replication, transcription, and virion morphogenesis [30,31,34,35]. However, the precise mechanism remained unclear. In our study, we successfully mapped key phosphorylated residues on H5 that were critical for condensate formation. We found that phosphorylation at two serine residues S127 and S130 not only contributed to H5 condensate assembly but also are required for viral factory formation and enhanced viral replication. Remarkably, mutation of S127 and S130 profoundly compromised H5’s biological function during VACV infection without affecting its synthesis or subcellular localization, highlighting the sensitivity of viral replication to specific post-translational modifications. The fact that alteration of only two residues completely abolished replication of such a large DNA virus underscores H5’s central role as a multifunctional hub. Importantly, H5 orthologs from VACV, LSDV, and MPXV were also able to form condensates, indicating that this LLPS capability is conserved among poxviruses (Fig 9).

The ability of H5 to form condensates within infected cells is driven by its N-terminal IDR, which comprises 134 amino acids. IDRs are crucial for proteins that mediate LLPS, often due to their structural flexibility and capacity to engage in multiple weak interactions that facilitate condensate formation [3]. In poxvirus-infected cells, viral factories are DNA-rich compartments that concentrate viral components to support efficient replication, yet the nature of these membrane-less organelles remains enigmatic [24–26,36]. Our data indicated that impairing H5’s phosphorylation at S127 and S130 substantially disrupted viral factory formation, even though H5 synthesis and general localization were largely unaffected. This disruption led to the cessation of viral DNA replication and overall viral replication, highlighting the mechanism by which H5’s scaffold-like interactions, nucleic-acid binding, and phosphorylation-dependent activity converge to organize viral replication compartments [29–35,49]. These findings provide a mechanistic link between H5-mediated condensates and the formation of functional viral factories, consistent with studies of other viruses where phase separation concentrates replication machinery and regulatory factors [19–23,39–41]. Nevertheless, disruption of H5’s phosphorylation sites may also compromise its interactions with other cellular proteins, which are required for H5’s LLPS property. Attempts to resolve H5’s structure experimentally were unsuccessful; therefore, we used AlphaFold3 to predict its structure, which confirmed the flexible nature of the IDR and delineated the boundary between the IDR and the C-terminal domain [42,43]. This structural insight was crucial for designing truncation mutants and interpreting their effects on condensate and factory formation.

Post-translational modifications, particularly phosphorylation, play a crucial role in regulating LLPS by modulating protein-protein and protein-nucleic-acid interactions [13–17]. In the context of poxvirus infection, the viral kinase B1 and host kinases VRK1/2 perform overlapping phosphorylation functions that impact viral replication [32,48,49,53]. One of their major substrates appears to be H5, whose phosphorylation is essential for condensate formation and viral factory assembly [29,31,47]. VRK1 is primarily nuclear, whereas VRK2 localizes to the cytoplasm and nuclear membrane, making it a more likely candidate for cytoplasmic H5 phosphorylation. Indeed, suppression of VRK2 in transfected cells abrogated H5’s ability to form condensates, suggesting VRK2 contributes to H5-LLPS formation. However, during actual viral infection, VRK2 knockdown alone did not affect H5 condensate formation or viral replication, indicating functional redundancy with other kinases such as virus B1. Beyond these kinases, our data suggest that additional host factors, such as MAPK15, may also contribute to H5 phosphorylation. It is possible that pharmacological inhibition by dihydrobaicalein may act on VRK2, thereby blocking H5 phosphorylation and subsequent condensate formation. However, because dihydrobaicalein also inhibits PLK1 and PLK2—and given that PLK1 is known to contribute to VACV replication—a minor off-target effect on viral replication cannot be excluded [54,55]. Collectively, these observations, together with previous reports, underscore that H5—an essential multifunctional protein—has evolved to exploit multiple host and viral kinases to sustain its phosphorylation, potentially driving LLPS and supporting viral replication.

To identify the key amino acid sites for H5 phosphorylation, we screened all possible residues on H5 and narrowed the critical residues down to S127 and S130. Mutations of only one residue had a partial effect, whereas the combination completely abolished H5’s ability to form condensates. Recent studies on MPXV H5 phosphorylation are consistent with our findings [47], showing that combined mutation of three phosphorylation-related residues on the α2 helix of MPXV-H5 (S134, S137, and S140, corresponding to S127, S130, and S133 of VACV-H5) impairs H5 binding to dsDNA, suggesting that phosphorylation enhances dsDNA binding and contributes to condensate formation. Nevertheless, it is important to point out that H5 also functions as a scaffold protein, which interacts with DNA and possibly multiple partner proteins within viral factories, such as the E9–A20–D4 DNA replication complex [27–29,34,35]. Mutations at S127 and S130 may inhibit condensate formation by disrupting these protein–protein interactions, or conversely, impaired LLPS could weaken H5’s interaction with other partner proteins, ultimately blocking viral DNA replication and virus replication. In addition, it is worth noting that loss of phosphorylation at S127 and S130 may alter H5’s conformation, thereby weakening its interactions with as-yet-unidentified cellular proteins and ultimately impairing LLPS formation.

Although H5 alone was able to form condensates when transfected in cells, cell-free assays revealed that the presence of only H5 and dsDNA was insufficient. In the cell-free system, dsDNA was able to bind to H5 and induce condensate-like puncta in the absence of other cellular components; however, these condensates did not exhibit FRAP. The addition of cell lysates induced H5 to adopt an LSPS (liquid-solid phase separation) state, a phenomenon reminiscent of that of purified cGAS, which undergoes LLPS in cells but shifts to LSPS when bound to DNA in a cell-free context [51]. These findings suggest that LLPS of H5 depends on additional cellular factors. Through mass spectrometry, we identified XRCC5/6 as possible H5 binding partners, and addition of purified XRCC5 or XRCC6 to the H5–dsDNA system partially restored LLPS property of H5, indicating that additional host factors may contribute. Notably, XRCC5/6 (Ku70/Ku80) form a ring-shaped heterodimer that binds double-stranded DNA ends with high affinity in a sequence-independent manner and can slide along DNA, while also recruiting DNA-PKcs to mediate non-homologous end joining (NHEJ). Such ‘end-bridging/clamping’ and multivalent nucleic acid-binding properties could, in principle, provide additional adhesion sites and dynamic exchange interfaces for H5–dsDNA condensates, thereby facilitating the restoration of their liquid-like characteristics [56,57]. In addition, the Ku complex has been implicated in interactions with viral replication compartments or genomes during infections with various DNA viruses and has been reported to participate in cytosolic DNA sensing, providing a plausible biological basis for its potential role in organizing the DNA-rich replication factories of poxviruses [56,58]. However, its precise mode of action in this context remains to be experimentally determined. It also remains unclear whether other viral proteins contribute to this process. Moreover, whether disruption of H5 condensates impairs its interactions with viral proteins—particularly those localized to viral factories, such as the DNA polymerase complex—is largely unknown. Further studies are needed to precisely define the host and viral components that contribute to H5 condensate formation and viral factory maturation. Collectively, these findings highlight H5 as a multifunctional phosphoprotein that functions as a nucleic acid–binding protein and a scaffold for viral replication and transcription complexes. They further demonstrate that phosphorylation at S127 and S130 serves as a critical regulatory step for LLPS formation and viral factory assembly, with broad implications for coordinating interactions with both viral and host factors. These insights position H5 phosphorylation as a promising target for antiviral intervention.

A key aspect of this study is the role of H5 in the formation of viral replication factories, which are primarily located in the cytoplasm of infected cells for poxvirus infection [25]. The formation of replication factories is crucial for efficient viral replication and assembly, as they concentrate viral and host factors within specific cytoplasmic regions [25]. Previous studies have highlighted the importance of condensates formation in organizing viral components into functional compartments [19,20,41]. In another study, H5 was predicted as the only candidate for the scaffold protein of LLPS [36]. H5 localizes to puncta when heterologously expressed in mammalian cells and is essential for the formation of prereplication foci [31]. In the structural analysis of MPXV DNA polymerase, H5 is also considered to serve as a scaffold for the polymerase complex to bind DNA, highlights its role in the formation of the DNA replication complex [52]. Previous studies have shown that VACV B1 and VRK2 kinases promote the formation of viral replication factories through phosphorylation-dependent inhibition of VACV B12 [49]. Our research proposes another possibility that B1 and VRK2 may regulate the binding of H5 to dsDNA in a phosphorylation-dependent manner, mediating H5-driven LLPS and further promoting the formation of viral replication factories. The ability of H5 to form condensates is critical for establishing these replication factories, effectively compartmentalizing the replication mechanism. This finding aligns with research on other viral systems, such as rabies and influenza viruses, in which condensates formation enhances viral transcription and replication efficiency [19,20,41]. However, the dynamic characteristics of these condensates and their impact on spatial and temporal regulation of viral replication remain unclear. Future research could focus on real-time cell imaging techniques to observe the dynamic changes of H5 condensates during the viral replication cycle, providing deeper insights into how these structures contribute to replication factory efficiency and regulation, and informing antiviral strategies aimed at disrupting these critical interactions and inhibiting viral replication at multiple stages of the viral lifecycle.

The conservation of LLPS properties among H5 homologs across different poxviruses suggests that this mechanism may play a critical role in the viral life cycle. This evolutionary conservation underscores its significance in poxvirus biology and provides a strong rationale for developing targeted, broad-spectrum antiviral strategies. Disrupting H5’s ability to form condensates could hinder viral replication. Similar approaches have been proposed for other viruses, such as targeting condensates formation of the SARS-CoV-2 nucleocapsid protein to inhibit viral replication [21–23]. Future research could focus on high-throughput screening of chemical libraries to identify small molecules or peptides that specifically disrupt H5 condensates and assess their efficacy and safety in preclinical and clinical attempts. Additionally, structure-based drug design methods using detailed structural information of H5 and its condensates could aid in developing effective and selective inhibitors. Evaluating the potential off-target effects and toxicity of these inhibitors in mammalian cells is crucial for advancing these therapeutic candidates to clinical application.

## Materials and methods

### Ethics statement

All animal experiments were jointly performed by Yantai TeK Biotechnology Co. and the Lanzhou Veterinary Research Institute, in accordance with the relevant regulations and guidelines for the care and use of laboratory animals. All experimental procedures were reviewed and approved by the Animal Welfare and Ethics Committee of the Lanzhou Veterinary Research Institute, Chinese Academy of Agricultural Sciences (Approval No. 2025–030).

### Cell culture and generation of cell lines

Human A549 cells (ATCC CCL-185), HEK-293T cells (ATCC CRL-3216) and BS-C-1 cells (ATCC CCL-26) were maintained at 37 °C in a humidified 5% CO_2_ incubator. A549 and BS-C-1 cells were cultured in DMEM/F-12 (1:1; Solarbio) supplemented with 10% heat-inactivated fetal bovine serum (FBS, Gibco), 1% penicillin-streptomycin (100 U/mL penicillin, 100 μg/mL streptomycin; Beyotime) and 2 mM L-glutamine (Gibco). HEK-293T cells were cultured in high-glucose DMEM (Gibco) with the same supplements. Cells were passaged every 2 ~ 3 days using 0.25% trypsin-EDTA (Gibco) and were used at passage numbers that ensured exponential growth.

The doxycycline-inducible A549^H5-eGFP^ cell line was generated by lentiviral transduction. The VACV H5 open reading frame (strain WR) with eGFP cloned at its C-terminal was cloned into the pLVX-TetOne-Puro vector (Takara #631849) and the sequence was verified by Sanger sequencing. Lentiviral particles were produced by transient transfection of HEK-293T producer cells (70–80% confluence) with the transfer plasmid (pLVX-H5-eGFP), the packaging plasmid psPAX2 (Addgene #12260) and the envelope plasmid pMD2.G (Addgene #12259) using Lipofectamine 3000 (Thermo Fisher) according to standard protocols provided by the manufacturer. Supernatants containing lentivirus were collected 48 hours post-transfection, clarified by low-speed centrifugation (500 × g, 5 min) and filtered through 0.22 μm PES filters to remove cell debris. A549 cells were then transduced with viral supernatant (undiluted or with serial dilutions to optimize multiplicity) in the presence of 5 μg/mL polybrene (Sigma) for 16 ~ 24 hours, and then replaced with fresh medium. Transduced cells were then selected with 1 μg/mL puromycin for 7 days and maintained in medium containing 1 μg/mL puromycin. For inducible expression, cells were treated with 2 μg/mL doxycycline for 24 hours prior to assays. H5-eGFP expression was confirmed by Western blotting using an anti-H5 antibody (1:1,000) and β-actin as loading control. Lentiviral production and transduction procedures followed established recommendations for safe and efficient lentiviral vector generation and titration [59,60].

### Antibodies

Rabbit polyclonal antibody against vaccinia virus (VACV) WR strain and mouse monoclonal antibody against VACV I3 protein were kindly provided by Dr.Bernard Moss (NIH) and have been described previously [61,62]. Mouse anti-β-actin antibody (#AA128, Beyotime Biotechnology) was used as a loading control in Western blotting (WB, 1:5,000). Mouse anti-eGFP antibody (#AG281, Beyotime Biotechnology) was used for WB (1:2,000). Horseradish peroxidase (HRP)-conjugated goat anti-mouse IgG (#A0216, Beyotime Biotechnology) and goat anti-rabbit IgG (#A0208, Beyotime Biotechnology) were used as secondary antibodies in WB (1:10,000). For immunofluorescence (IF), Alexa Fluor 488–conjugated goat anti-mouse IgG (#A32723, Thermo Fisher Scientific) and Alexa Fluor 555–conjugated goat anti-rabbit IgG (#A32732, Thermo Fisher Scientific) were applied at 1:1,000. All antibodies were diluted in 3% bovine serum albumin (BSA) in PBS-T (0.1% Tween-20) depending on the assay.

### Generation of recombinant VACV (VACV-WR^ΔH5^ with mCherry insertion)

To generate VACV-WR^ΔH5^, approximately 500 bp sequences immediately upstream (left arm) and downstream (right arm) of the VACV H5 ORF were PCR-amplified from VACV-WR genomic DNA using Phusion High-Fidelity DNA Polymerase (#M0530L, New England Biolabs). The mCherry ORF under control of the VACV p11 early/late promoter was synthesized and inserted between the two flanks to create the recombination cassette via overlap-extension PCR [63–65]. The p11 promoter is a well-characterized late gene promoter that drives robust transgene expression during the late phase of infection, and its activity is largely independent of the insertion locus, as recently shown in MVA vectors [66–68]. The final cassette structure was: left arm–p11 promoter–mCherry–right arm.

A549^H5-eGFP^ cells pre-induced with 2 μg/mL doxycycline for 24 hours were infected with parental VACV-WR at a multiplicity of infection (MOI) of 3 PFU/cell for 1 hour at 37 °C. Following adsorption, the cells were transfected with 1 ~ 2 μg of the linearized recombination cassette using Lipofectamine 3000 (Thermo Fisher) in Opti-MEM (Gibco), following the manufacturer’s guidelines. Cells were fixed with overlay medium (1.5% carboxymethylcellulose in DMEM with 2% FBS) at 24 ~ 48 hours post-infection to allow plaque formation and visual assessment under fluorescence (mCherry).

Recombinant plaques expressing mCherry were identified by fluorescent microscopy, picked with pipette tips, and subjected to at least three rounds of plaque purification in A549^H5-eGFP^ cells. Viruses were harvested by repeated freeze–thaw cycles of infected cells, clarified by low-speed centrifugation (500 × g, 5 min), and stored at –80 °C. The deletion of H5 and insertion of mCherry were confirmed by PCR using primers flanking the H5 locus and Sanger sequencing. A primer internal to H5 was also used to demonstrate its absence. Viral titers were determined by standard plaque assay on A549^H5-eGFP^ cells [65].

### Fluorescence recovery after photobleaching

A549 cells were infected with VACV-WR or transfected with H5-eGFP, and FRAP assays were performed using a Nikon A1 HD25 confocal microscope equipped with a 100 × oil-immersion objective (NA 1.45). Pre-bleach images were acquired at 5 and 10 seconds before bleaching. A defined region of interest (ROI) was photobleached twice for 13 seconds using a 488 nm laser at 100% power. Time-lapse images were then collected every 5 seconds for 2 minutes post-bleach. Fluorescence intensity within the ROI was measured and normalized to pre-bleach levels using NIS-Elements software. FRAP data were analyzed to assess the mobility and recovery kinetics of the fluorescent proteins.

### Confocal microscopy

Human A549 cells were cultured on glass coverslips and infected with the indicated viruses at 3 PFU/cell. At various hours post-infection, cells were fixed with 4% paraformaldehyde (PFA) for 15 min at room temperature, permeabilized with 0.5% Triton X-100 for 15 min, and blocked with 3% BSA in 1 × phosphate-buffered saline (PBS) for 30 min. Primary antibodies were diluted in 3% BSA-PBS and incubated with cells overnight at 4°C. After washing with PBS, cells were incubated with secondary antibodies conjugated to Alexa Fluor 488, 555, or 647 for 1 hour at room temperature in the dark. Nuclei and viral factories were stained with Hoechst 33342, and cells were washed thoroughly before mounting on slides using ProLong Diamond Antifade reagent (Thermo Fisher Scientific). Images were acquired on a Nikon A1 HD25 confocal microscope and processed using NIS-Elements software.

### SDS-PAGE and Western blotting analysis

For reducing SDS-PAGE, human A549 cells were washed once with ice-cold PBS and lysed in wells with 150 µL 1 × RIPA lysis buffer (Beyotime Biotechnology) containing 1 × PMSF (Beyotime Biotechnology) in each well of a 6-well plate on ice. 5 × protein loading buffer containing 0.05 M DTT (Beyotime Biotechnology) was added to the cell lysate and boiled for 10 minutes.

For non-reducing SDS-PAGE, human A549 cells were lysed in wells with 150 µL 1 × NP40 lysis buffer (Beyotime Biotechnology) containing 1 × PMSF (Beyotime Biotechnology) in each well of a 6-well plate on ice. 5 × non-reducing protein loading buffer (Beyotime Biotechnology) without reducing agents was added to the cell lysate.

Proteins were resolved on 4–20% FastPAGE precast gels (Tsingke Biotechnology) and transferred to nitrocellulose membranes in a Trans-Blot Turbo machine (Bio-Rad). Membranes were blocked with 5% nonfat milk dissolved in Tris-buffered saline (TBS) for 1 hour at RT and then incubated with primary antibodies diluted 1 × TBS with 0.1% Tween 20 (TBST) containing 5% nonfat milk overnight at 4 °C. The membranes were washed three times with 1 × TBST and incubated with a secondary antibody conjugated with horseradish peroxidase diluted 1:5,000 in 1 × TBST containing 5% nonfat milk for 1 hour at RT. ECL signals were detected with SuperSignal West Dura substrates (Thermo Fisher Scientific) and visualized by Image J.

### Generation of H5 monoclonal antibody

The expressing plasmid encoding MBP-H5 conjugated with IL-2 signal peptide and 6 × his tag in its N-terminus was constructed, MBP-H5 was produced in HEK-293T cells and purified as reported before [69]. The protein was further purified on a Superdex 75 column (Cytiva). New Zealand white rabbits were immunized with purified MBP-H5 protein, and the rabbits received another 3 booster injections at 2-week intervals. Rabbit splenocytes were harvested using a Tissuelyser II sample disrupter (Qiagen). The cells were then pushed through a cell strainer to generate a single-cell suspension before being used for FACS or frozen in 10% DMSO in FCS. B cells were stained by anti-rabbit CD45R-FITC and MBP-H5 labeled with PE and further isolated from splenocytes by FACS. FACS was performed on a BD FACS ARIA III with single VACV H5-specific IgG^+^ B cells being deposited into the well of a 96-well PCR plate. After sorting single B cells into a 96-well plate containing 4 μL of lysis buffer in each well, cDNA generation, multiple PCR steps, and Sanger sequencing were performed as reported before for isolating V_H_ and V_L_ antibody genes [70]. Moreover, the expressing plasmids encoding antibody V_H_ gene and V_L_ gene were constructed respectively. The antibody heavy and light chain plasmids were transformed into HEK-293T cells to express H5 antibodies followed by further purification. The animal experiments described above were jointly performed by Yantai TeK Biotechnology Co. and the Lanzhou Veterinary Research Institute, in accordance with relevant regulations and guidelines for animal experimentation, and were approved by the Animal Welfare and Ethics Committee of the Lanzhou Veterinary Research Institute, Chinese Academy of Agricultural Sciences (Approval No. 2025–030).

### Mass spectrometry analysis of phosphorylated H5 and host interacting partners

A549 cells were infected with VACV-WR at 3 PFU/cell for 12 hours. Cells were then lysed using immunoprecipitation lysis buffer. To reduce non-specific binding, the cell lysate was first incubated with mouse IgG magnetic beads for 2 hours at 4°C. For phosphorylation analysis, lysates were incubated with magnetic beads pre-incubated with anti-H5 monoclonal antibody for 18 hours at 4°C, followed by five washes with TBS to remove unbound proteins. The immunoprecipitated H5 proteins were analyzed by mass spectrometry (MS), and phosphorylated peptides were identified based on the characteristic neutral loss of phosphoric acid (−98 Da) in the MS2 spectrum, with corresponding y or b fragment ions indicating serine phosphorylation sites [71].

To identify host proteins interacting with H5, the same IP-MS strategy was applied. To minimize the potential impact of viral DNA replication on the results, cells were treated with 10 µM Ara-C during infection. After immunoprecipitation with magnetic beads pre-incubated with anti-H5 monoclonal antibody and repeated washes (five times), the bound host proteins were eluted and subjected to LC-MS/MS analysis using a protocol described previously [72].

### Protein expression, purification, and preparation

For expression and purification of H5, the A549^H5-eGFP^ cell line was treated with 2 µg/mL doxycycline for 48 h to induce H5-eGFP expression, while 6 × his-flag-XRCC5 and 6 × his-flag-XRCC6 proteins were expressed by transfecting HEK-293T cells grown in suspension with plasmid pRK5 (Addgene # 99723) containing the ORF of XRCC5/6 cloned from cDNA of A549 cells. HEK-293T cells were transiently transfected with the corresponding plasmids using BeyoPEI Transfection Reagent (Beyotime Biotechnology) and incubated for 96 hours at 37 °C, 8% CO_2_, and 120 rpm until reaching a density of approximately 5 × 10^6^ cells/mL. Cells expressing H5-eGFP or XRCC5/6 were collected by centrifugation, washed twice with PBS, and lysed in 1 × Western and IP lysis buffer (20 mM Tris-HCl pH 7.4, 150 mM NaCl, 2 mM EDTA, 1% Triton X-100) containing protease inhibitor cocktail (CoWin Biosciences) and phosphatase inhibitor cocktail (Beyotime Biotechnology) on ice. Lysates were centrifuged at 15,000 × g for 20 min at 4 °C to remove cell debris. The protein-containing supernatant was exchanged into binding buffer (20 mM NaH_2_PO_4_, 0.5 M NaCl, pH 7.4) using Amicon Ultra Centrifugal Filter Units (Merck Millipore) and loaded onto Ni-NTA affinity columns (GenScript). Columns were washed with washing buffer (20 mM NaH_2_PO_4_, 0.5 M NaCl, 10 mM imidazole, pH 7.4), and proteins were eluted with elution buffer (20 mM NaH_2_PO_4_, 0.5 M NaCl, 500 mM imidazole, pH 7.4). Finally, imidazole was removed and proteins were concentrated and resuspended in storage buffer (50 mM Tris-HCl pH 7.4, 500 mM NaCl, 2 mM MgCl_2_) to prevent unwanted phase separation [73].

### Phase separation assays and FRAP in a cell-free system

Phase separation of purified H5-eGFP protein was analyzed in a cell-free system using Glass Bottom Cell Culture Dishes (NEST). Purified H5-eGFP protein was resuspended in phase separation buffer (50 mM Tris-HCl pH 7.4, 175 mM NaCl, 2 mM MgCl_2_, 1 mM ATP, 0.5 mM TCEP, 2.5% PEG 8000) optimized for promoting LLPS. For droplet formation, H5-eGFP (5–20 μM) was mixed with Cy3-labeled dsDNA (sequence as described previously) or partner proteins at 25 °C in a total reaction volume of 100 μL [50]. Protein:DNA or protein:protein molar ratios were adjusted to assess concentration-dependent droplet formation. Reactions were gently mixed and immediately imaged. Fluorescence imaging was performed on a Nikon A1 HD25 confocal microscope at 25 °C. The fluorescence of eGFP was excited at 488 nm and Cy3 at 552 nm. Droplet size, number, and morphology were quantified using NIS-Elements software.

FRAP experiments were performed to evaluate molecular dynamics within the phase-separated droplets. Each experimental condition was analyzed in three independent biological replicates, and three individual droplets were selected for photobleaching in each replicate. Droplets containing H5-eGFP, Cy3-dsDNA, or XRCC5/6 were photobleached using 30% maximum power of the 488 nm laser (or equivalent for other fluorophores), and recovery was recorded at 5 s intervals for ~120 s. Fluorescence intensities were normalized to pre-bleach values to analyze recovery kinetics, providing information on droplet fluidity and molecular mobility characteristic of LLPS.

### Determination of virus yield

BS-C-1 or A549^H5-eGFP^ cells were seeded in 24-well plates. For experiments involving A549^H5-eGFP^, cells were pretreated with 2 μg/mL doxycycline for 24 hours to induce H5 expression. Cells were then infected with viruses at an MOI of 0.03 PFU/cell in DMEM/F12 supplemented with 2.5% FBS and 1% penicillin/streptomycin for 2 hours. After adsorption, cells were washed twice with 1 × PBS and incubated in fresh medium for 48 hours. Viruses were harvested by scraping the cells and subjected to three freeze-thaw cycles to release viral particles.

For viral titration, duplicate monolayers of BS-C-1 or doxycycline-induced A549^H5-eGFP^ cells were infected with serially diluted virus stocks for 2 hours. The inoculum was removed, and cells were overlaid with medium containing complete DMEM containing 2.5% FBS and 0.5% methylcellulose. After 48 hours, cells were fixed with 1% crystal violet in 20% ethanol, plaques were counted, and average titers from triplicate wells were calculated.

### Immunoprecipitation and dsDNA-H5 binding assay

Human A549 cells were first washed with ice-cold PBS and processed according to the downstream assay. For cellular H5-interacting protein immunoprecipitation, cells were lysed in 150 µL per well of 1 × Western and IP lysis buffer containing 1 × PMSF on ice in 6-well plates. Lysates were clarified by centrifugation at 15,000 × g for 15 min at 4 °C, then incubated with control magnetic beads or eGFP-Trap magnetic beads at 4 °C for 18 h with gentle rotation to enrich H5-eGFP and associated host proteins.

To assess cellular dsDNA-H5 binding, cells were washed twice with ice-cold PBS and rapidly frozen in liquid nitrogen to preserve protein-DNA interactions. Frozen cells were lysed in 150 µL TAP buffer per well (50 mM Tris-HCl pH 7.5, 100 mM NaCl, 5% glycerol, 0.2% Nonidet-P40, 1.5 mM MgCl_2_) containing protease inhibitors on ice. Streptavidin-trap magnetic beads were preincubated with or without biotinylated dsDNA (5’-TACAGATCTACTAGTGATCTATGACTGATCTGTACATGATCTACA-3’) in TAP buffer at 4 °C for 1 hour, and clarified lysates were then incubated with the preloaded beads overnight at 4 °C [50]. Non-specifically bound proteins were removed by multiple washes with TAP buffer followed by detergent-removal washes.

For dsDNA-H5 binding in a cell-free environment, purified H5-eGFP protein was resuspended in store buffer (50 mM Tris-HCl pH 7.4, 500 mM NaCl, 2 mM MgCl_2_) to prevent unwanted phase separation and incubated with streptavidin-trap beads preloaded with or without dsDNA-biotin at 4 °C overnight [73]. For all assays, bound proteins were eluted by boiling with 30 µL of LDS sample buffer containing 100 mM DTT, resolved on 4–20% FastPAGE precast gels, and analyzed by Western blotting analysis according to the procedure described earlier in the Methods section.

### Quantification of viral genomic DNA by quantitative PCR

Monolayers of A549 cells in 24-well plates were infected with the indicated viruses at 3 PFU/cell for 1 hour in triplicate wells. Following infection, cells were washed twice with ice-cold 1 × PBS to remove unbound virus and incubated in complete DMEM/F-12 medium. At 6 hours post-infection, total cellular DNA was extracted using the DNeasy Blood & Tissue DNA mini kit (Qiagen) according to the manufacturer’s instructions, and DNA concentration and purity were assessed using a NanoDrop spectrophotometer (Thermo Fisher Scientific). Equal amounts of DNA (1 µg per sample) were serially diluted and subjected to quantitative PCR using gene-specific primers for the viral E11 gene (forward: 5’-ACGACGATGTATGTGCCTC-3’; reverse: 5’-CAAACAGCAAGGTTCGTCA-3’) and the host GAPDH gene (forward: 5’-ACCACCCTGTTGCTGTAGCCAA-3’; reverse: 5’-GTCTCCTCTGACTTCAACAGCG-3’) as an internal control. Quantitative PCR reactions were performed using a SYBR Green-based detection system with the following cycling conditions: initial denaturation at 95 °C for 3 min, followed by 40 cycles of 95 °C for 10 s and 60 °C for 30 s. All reactions were run in triplicate, and the specificity of amplification was verified by melt-curve analysis. Relative viral genomic DNA abundance was calculated using the ΔΔCt method, normalized to GAPDH, and represented as fold change compared to mock-infected controls.

### Statistics and reproducibility

The statistical analysis was conducted with Prism 10 (GraphPad). Differences were calculated using two-sided Student’s t test as specified in the figure legends. The bar plots of data represent the means ± SD (standard deviation) and the threshold for statistical significance was considered upon p < 0.05. All experiments were repeated at least three times. All statistical details of experiments were included in the figure legends.

## Supporting information

S1 Fig Identification of H5 monoclonal antibody and observation of H5 morphology in cells. (A) A549 cells were infected with WR^WT^ at 0.1 PFU/cell or transfected with H5-Flag. After 24 hours, cells were lysed and then cellular proteins were analyzed by Western blotting. (B) A549 cells were infected with WR^WT^ at 3 PFU/cell or transfected with H5 or H5-eGFP. At 2 hpi or cells were transfected for 24 hours, cells were then fixed, permeabilized, blocked, and stained with primary antibodies to H5 followed by fluorescent conjugated secondary antibodies. Hoechst was used to stain DNA. Scale bars are shown at bottom. Data are mean ±SD (standard deviation). *n* = 3.
(DOCX)

S2 Fig eGFP fused to the C-terminus of H5 does not affect the biological properties of WR. A549 cells were infected with WR or WR^H5-eGFP^ at 0.01 PFU/cell. Viruses were harvested at 24, 48, or 72 hpi and quantified by plaque assay. Data are mean ±SD. *n* = 3. two-sided Student’s t test; ns, not significant.
(DOCX)

S3 Fig Homology of H5 between different poxviruses. The H5 sequences of 12 representative strains of different orthopoxviruses and LSDV were analyzed by Snapgene. The red arrows indicate the serines at positions 127 and 130 of H5. The green arrows indicate amino acids at positions 170 and 177 of H5 that are located on the dsDNA binding interface.
(DOCX)

S4 Fig Knockdown efficiency of VRK1/2 by siRNAs. (A and B) Three different siRNAs of VRK1 or VRK2 were transfected into A549 cells, and qPCR was used to measure the mRNA of VRK1 (A) or VRK2 (B) abundance 48 hours later. (C and D) A549 cells were transfected with the siNC, siVRK1–1, or siVRK2–2 for 48 hours, then infected with WR^H5-eGFP^ at 3 PFU/cell for 4 hours. qPCR was used to measure the mRNA of VRK1 (C) or VRK2 (D) abundance. Data are mean ±SD. n = 3.
(DOCX)

S5 Fig Knockdown of MAPK15 markedly inhibits the formation of H5 condensates. (A-C) siRNAs of MAPK3/14/15 were transfected into A549 cells, and qPCR was used to measure the mRNA of MAPK3 (A), MAPK14 (B) or MAPK15 (C) abundance 48 hours later. (D and E) A549 cells were transfected with the siNC, siMAPK3, siMAPK14 or siMAPK15 for 48 hours, then transfected with H5 for 24 hours. Fluorescence recovery was analyzed by FRAP (D), and relative fluorescence intensity versus time was recorded (E). Data are mean ±SD. n = 3.
(DOCX)

S6 Fig Identification of A549 cell line inducibly expressing H5. A549 cells were transfected with H5-eGFP, and A549 inducible expression cell lines (3B9 and 3B10) were added with doxycycline at a final concentration of 2 μg/mL. After 24 hours, cells were lysed and cellular proteins were analyzed by Western blotting.
(DOCX)

S7 Fig Representative FRAP analyses of H5–dsDNA condensates in the presence of XRCC5 or XRCC6. Purified H5-eGFP was mixed with Cy3-labeled dsDNA and XRCC5/6 exchanged into the same buffer system (50 mM Tris-HCl pH 7.4, 175 mM NaCl, 2 mM MgCl_2_, 1 mM ATP, 0.5 mM TCEP, 2.5% PEG 8000) at 25 °C to induce phase separation. Fluorescence recovery was analyzed by FRAP. Representative FRAP images showing two additional droplets from the same experiment as in Fig 6G.
(DOCX)

S1 Table The dataset used to build images in this article. (XLSX)

S1 Data The raw data of Western blotting and IF in this study. (PDF)
