# Supplementary material for: Poxvirus H5 mediates the formation of liquid-liquid phase separation condensates which promote virus factory assembly
Source: PLoS Pathog. 2025 Nov 20;21(11):e1013708. doi: 10.1371/journal.ppat.1013708 (PMC12633886; doi:10.1371/journal.ppat.1013708)
Supplement: S1 Fig — (A) A549 cells were infected with WRWT at 0.1 PFU/cell or transfected with H5-Flag. After 24 hours, cells were lysed and then cellular proteins were analyzed by Western blotting. (B) A549 cells were infected with WRWT at 3 PFU/cell or transfected with H5 or H5-eGFP. At 2 hpi or cells were transfected for 24 hours, cells were then fixed, permeabilized, blocked, and stained with primary antibodies to H5 followed by fluorescent conjugated secondary antibodies. Hoechst was used to stain DNA. Scale bars are shown at bottom. Data are mean ±SD (standard deviation). n = 3. (DOCX) [file ppat.1013708.s001.docx]

## S1 Fig. Identification of H5 monoclonal antibody and observation of H5 morphology in cells. (A) A549 cells were infected with WR^WT^ at 0.1 PFU/cell or transfected with H5-Flag. After 24 hours, cells were lysed and then cellular proteins were analyzed by Western blotting. (B) A549 cells were infected with WR^WT^ at 3 PFU/cell or transfected with H5 or H5-eGFP. At 2 hpi or cells were transfected for 24 hours, cells were then fixed, permeabilized, blocked, and stained with primary antibodies to H5 followed by fluorescent conjugated secondary antibodies. Hoechst was used to stain DNA. Scale bars are shown at bottom. Data are mean ±SD (standard deviation). n = 3.
