# Supplementary material for: Poxvirus H5 mediates the formation of liquid-liquid phase separation condensates which promote virus factory assembly
Source: PLoS Pathog. 2025 Nov 20;21(11):e1013708. doi: 10.1371/journal.ppat.1013708 (PMC12633886; doi:10.1371/journal.ppat.1013708)
Supplement: S2 Fig — A549 cells were infected with WR or WRH5-eGFP at 0.01 PFU/cell. Viruses were harvested at 24, 48, or 72 hpi and quantified by plaque assay. Data are mean ±SD. n = 3. two-sided Student’s t test; ns, not significant. (DOCX) [file ppat.1013708.s002.docx]

##

## S2 Fig. eGFP fused to the C-terminus of H5 does not affect the biological properties of WR. A549 cells were infected with WR or WR^H5-eGFP^ at 0.01 PFU/cell. Viruses were harvested at 24, 48, or 72 hpi and quantified by plaque assay. Data are mean ±SD. n = 3. two-sided Student’s t test; ns, not significant.
