# Supplementary material for: Poxvirus H5 mediates the formation of liquid-liquid phase separation condensates which promote virus factory assembly
Source: PLoS Pathog. 2025 Nov 20;21(11):e1013708. doi: 10.1371/journal.ppat.1013708 (PMC12633886; doi:10.1371/journal.ppat.1013708)
Supplement: S3 Fig — The H5 sequences of 12 representative strains of different orthopoxviruses and LSDV were analyzed by Snapgene. The red arrows indicate the serines at positions 127 and 130 of H5. The green arrows indicate amino acids at positions 170 and 177 of H5 that are located on the dsDNA binding interface. (DOCX) [file ppat.1013708.s003.docx]

##

## S3 Fig. Homology of H5 between different poxviruses. The H5 sequences of 12 representative strains of different orthopoxviruses and LSDV were analyzed by Snapgene. The red arrows indicate the serines at positions 127 and 130 of H5. The green arrows indicate amino acids at positions 170 and 177 of H5 that are located on the dsDNA binding interface.
