# Supplementary material for: Poxvirus H5 mediates the formation of liquid-liquid phase separation condensates which promote virus factory assembly
Source: PLoS Pathog. 2025 Nov 20;21(11):e1013708. doi: 10.1371/journal.ppat.1013708 (PMC12633886; doi:10.1371/journal.ppat.1013708)
Supplement: S4 Fig — (A and B) Three different siRNAs of VRK1 or VRK2 were transfected into A549 cells, and qPCR was used to measure the mRNA of VRK1 (A) or VRK2 (B) abundance 48 hours later. (C and D) A549 cells were transfected with the siNC, siVRK1–1, or siVRK2–2 for 48 hours, then infected with WRH5-eGFP at 3 PFU/cell for 4 hours. qPCR was used to measure the mRNA of VRK1 (C) or VRK2 (D) abundance. Data are mean ±SD. n = 3. (DOCX) [file ppat.1013708.s004.docx]

##
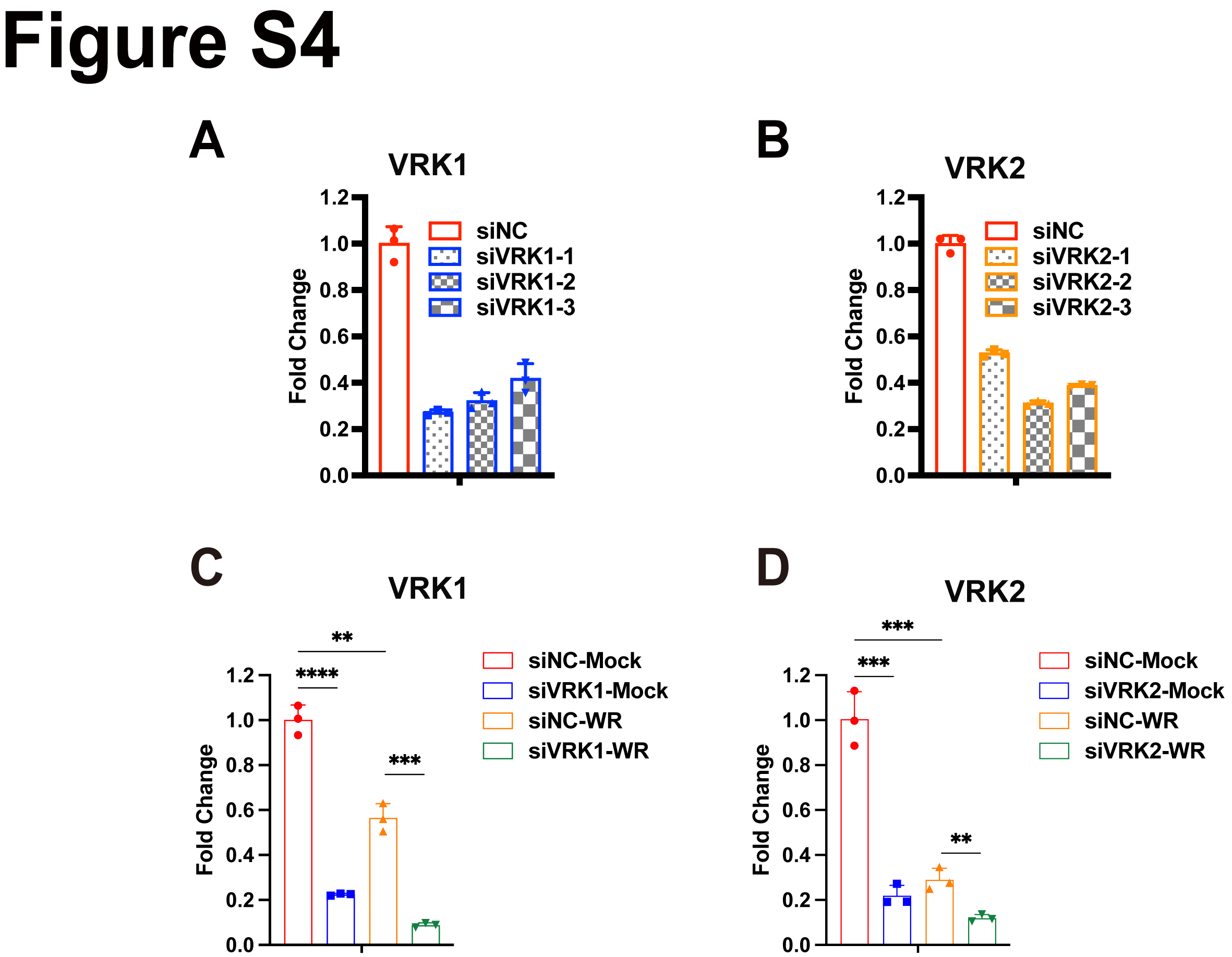


## S4 Fig. Knockdown efficiency of VRK1/2 by siRNAs. (A and B) Three different siRNAs of VRK1 or VRK2 were transfected into A549 cells, and qPCR was used to measure the mRNA of VRK1 (A) or VRK2 (B) abundance 48 hours later. (C and D) A549 cells were transfected with the siNC, siVRK1-1, or siVRK2-2 for 48 hours, then infected with WR^H5-eGFP^ at 3 PFU/cell for 4 hours. qPCR was used to measure the mRNA of VRK1 (C) or VRK2 (D) abundance. Data are mean ±SD. n = 3.
