# Supplementary material for: Poxvirus H5 mediates the formation of liquid-liquid phase separation condensates which promote virus factory assembly
Source: PLoS Pathog. 2025 Nov 20;21(11):e1013708. doi: 10.1371/journal.ppat.1013708 (PMC12633886; doi:10.1371/journal.ppat.1013708)
Supplement: S5 Fig — (A-C) siRNAs of MAPK3/14/15 were transfected into A549 cells, and qPCR was used to measure the mRNA of MAPK3 (A), MAPK14 (B) or MAPK15 (C) abundance 48 hours later. (D and E) A549 cells were transfected with the siNC, siMAPK3, siMAPK14 or siMAPK15 for 48 hours, then transfected with H5 for 24 hours. Fluorescence recovery was analyzed by FRAP (D), and relative fluorescence intensity versus time was recorded (E). Data are mean ±SD. n = 3. (DOCX) [file ppat.1013708.s005.docx]

##
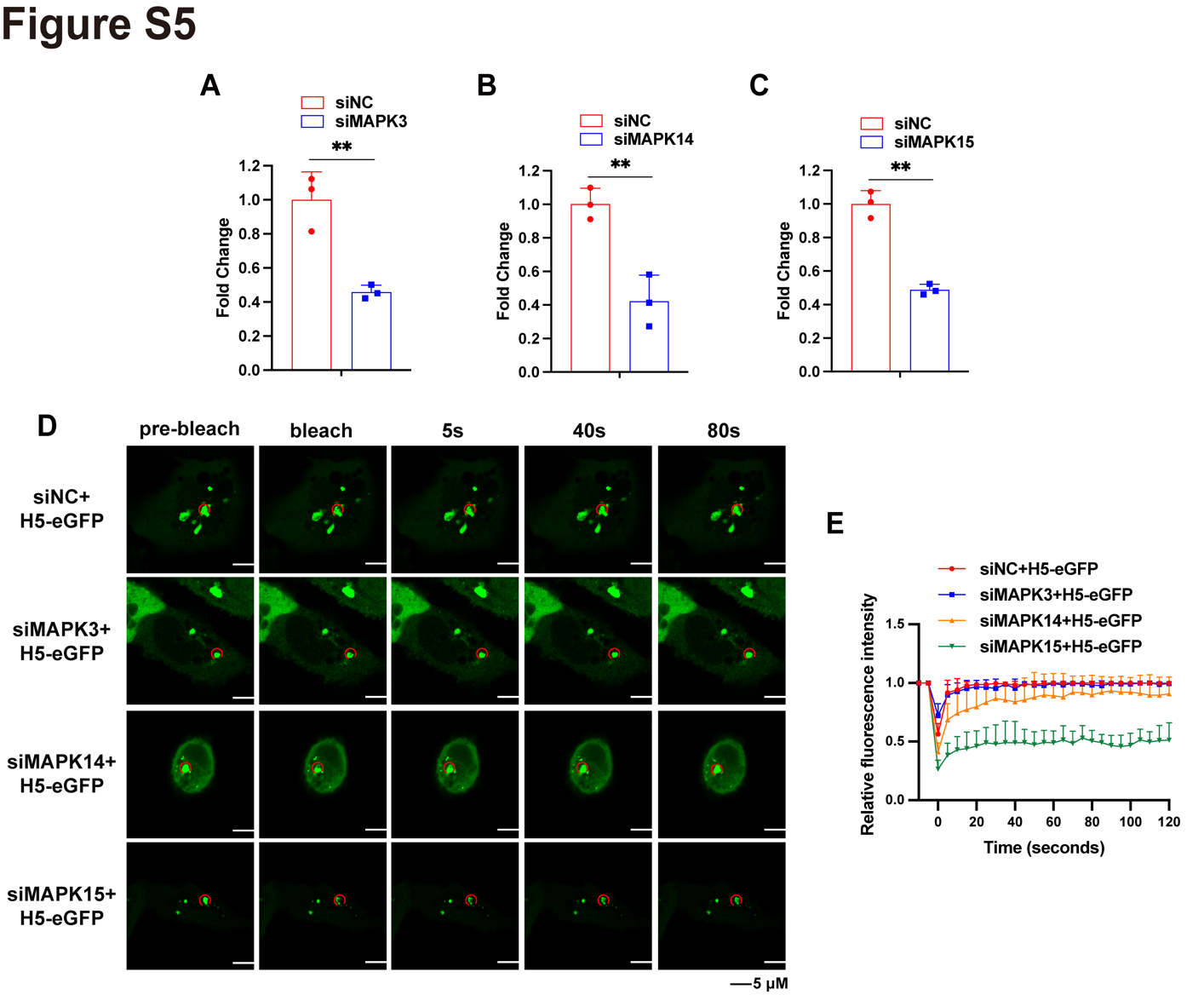


## S5 Fig. Knockdown of MAPK15 markedly inhibits the formation of H5 condensates. (A-C) siRNAs of MAPK3/14/15 were transfected into A549 cells, and qPCR was used to measure the mRNA of MAPK3 (A), MAPK14 (B) or MAPK15 (C) abundance 48 hours later. (D and E) A549 cells were transfected with the siNC, siMAPK3, siMAPK14 or siMAPK15 for 48 hours, then transfected with H5 for 24 hours. Fluorescence recovery was analyzed by FRAP (D), and relative fluorescence intensity versus time was recorded (E). Data are mean ±SD. n = 3.
