# Supplementary material for: Poxvirus H5 mediates the formation of liquid-liquid phase separation condensates which promote virus factory assembly
Source: PLoS Pathog. 2025 Nov 20;21(11):e1013708. doi: 10.1371/journal.ppat.1013708 (PMC12633886; doi:10.1371/journal.ppat.1013708)
Supplement: S6 Fig — A549 cells were transfected with H5-eGFP, and A549 inducible expression cell lines (3B9 and 3B10) were added with doxycycline at a final concentration of 2 μg/mL. After 24 hours, cells were lysed and cellular proteins were analyzed by Western blotting. (DOCX) [file ppat.1013708.s006.docx]

##
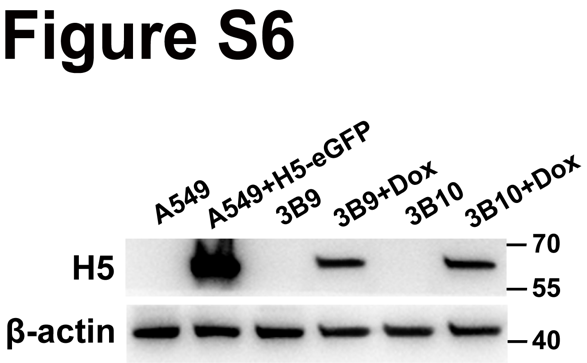


## S6 Fig. Identification of A549 cell line inducibly expressing H5. A549 cells were transfected with H5-eGFP, and A549 inducible expression cell lines (3B9 and 3B10) were added with doxycycline at a final concentration of 2 μg/mL. After 24 hours, cells were lysed and cellular proteins were analyzed by Western blotting.
