# Supplementary material for: Poxvirus H5 mediates the formation of liquid-liquid phase separation condensates which promote virus factory assembly
Source: PLoS Pathog. 2025 Nov 20;21(11):e1013708. doi: 10.1371/journal.ppat.1013708 (PMC12633886; doi:10.1371/journal.ppat.1013708)
Supplement: S7 Fig — Purified H5-eGFP was mixed with Cy3-labeled dsDNA and XRCC5/6 exchanged into the same buffer system (50 mM Tris-HCl pH 7.4, 175 mM NaCl, 2 mM MgCl2, 1 mM ATP, 0.5 mM TCEP, 2.5% PEG 8000) at 25 °C to induce phase separation. Fluorescence recovery was analyzed by FRAP. Representative FRAP images showing two additional droplets from the same experiment as in Fig 6G. (DOCX) [file ppat.1013708.s007.docx]

##
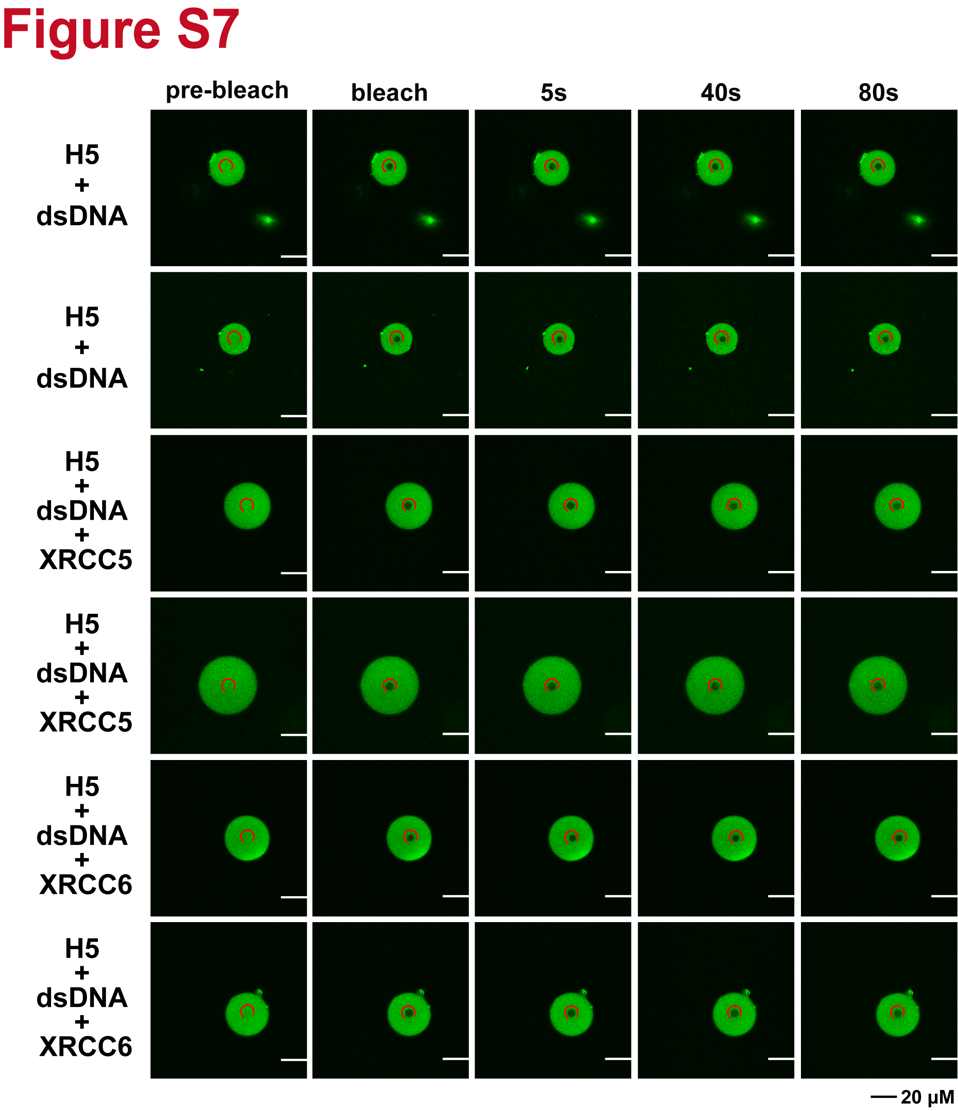


## S7 Fig. Representative FRAP analyses of H5–dsDNA condensates in the presence of XRCC5 or XRCC6. Purified H5-eGFP was mixed with Cy3-labeled dsDNA and XRCC5/6 exchanged into the same buffer system (50 mM Tris-HCl pH 7.4, 175 mM NaCl, 2 mM MgCl_2_, 1 mM ATP, 0.5 mM TCEP, 2.5% PEG 8000) at 25 ℃ to induce phase separation. Fluorescence recovery was analyzed by FRAP. Representative FRAP images showing two additional droplets from the same experiment as in Fig 6G.
