# Supplementary material for: Poxvirus H5 mediates the formation of liquid-liquid phase separation condensates which promote virus factory assembly
Source: PLoS Pathog. 2025 Nov 20;21(11):e1013708. doi: 10.1371/journal.ppat.1013708 (PMC12633886; doi:10.1371/journal.ppat.1013708)
Supplement: S1 Data — (PDF) [file ppat.1013708.s009.pdf]

Original Images for Blots and IF

Poxvirus H5 mediates the formation of liquid-liquid phase separation condensates which promote virus factory assembly

Junda Zhu, Zihui Zhang, Yongxiang Fang, Jian Xu, Zhimin Jiang, Hua Li, Shijie Xie, Kang Niu, Zhizhong Jing, Baifen Song, Wenxue Wu, Chen Peng

Fig 2C

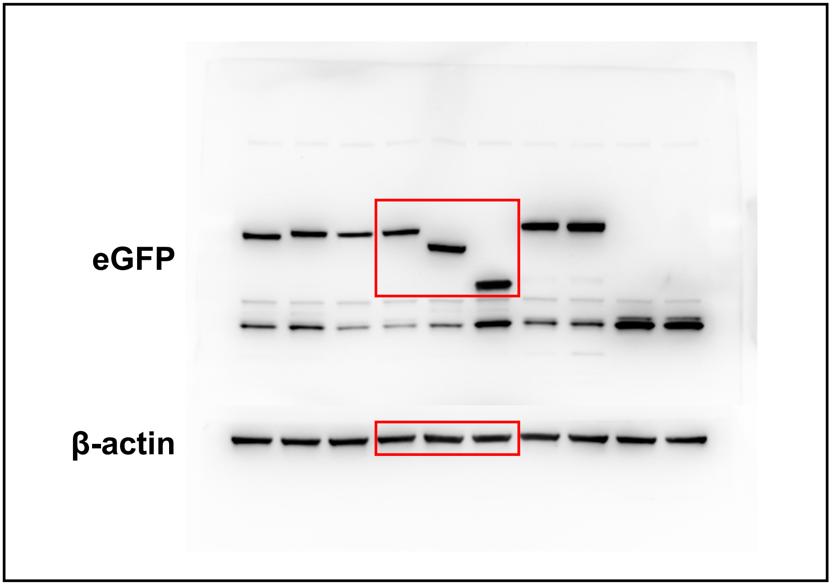

Fig 6A

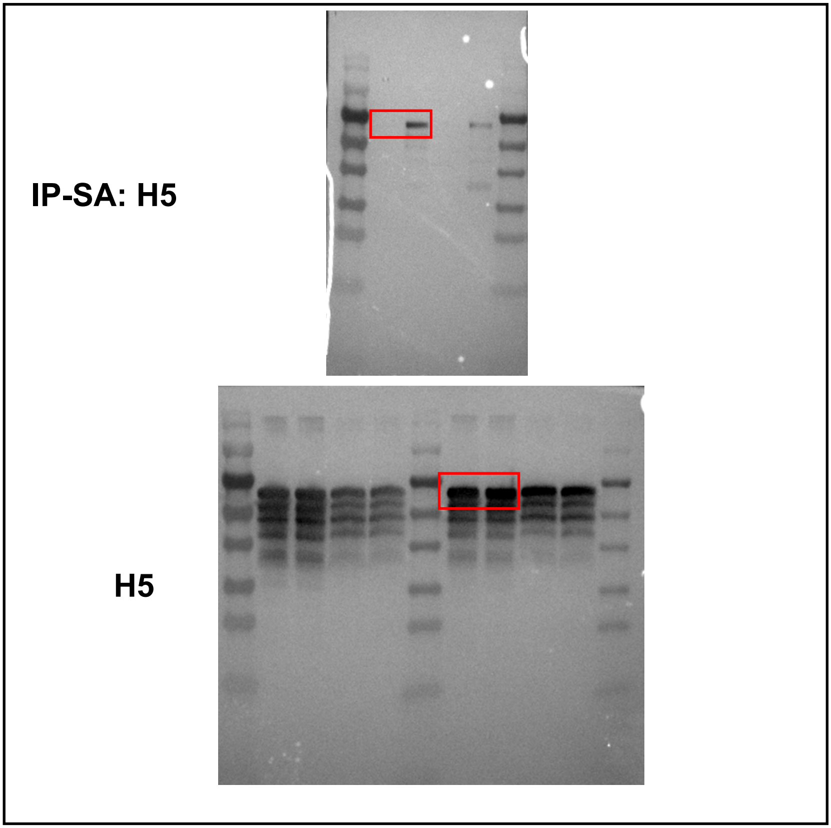

Fig 7A

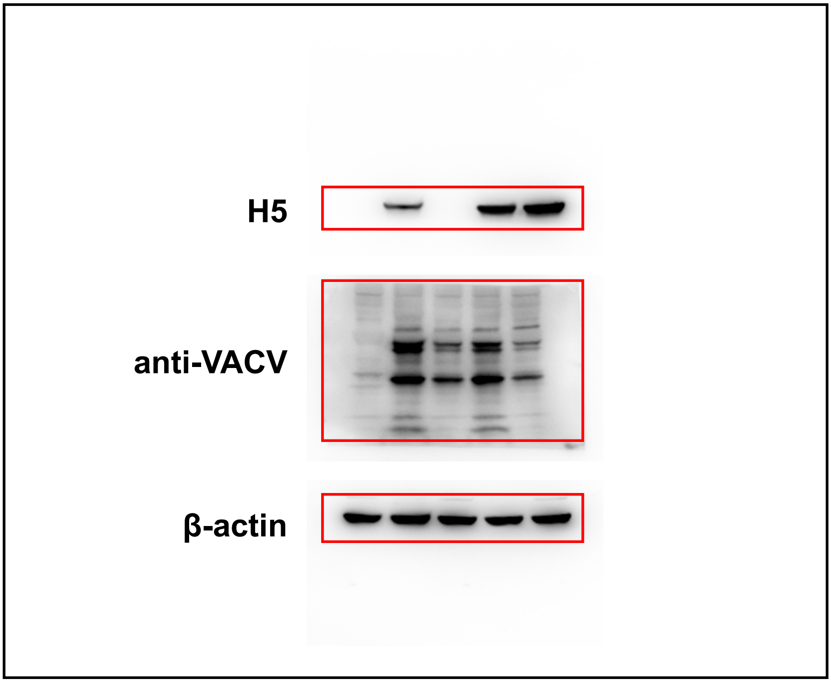

Fig 7D

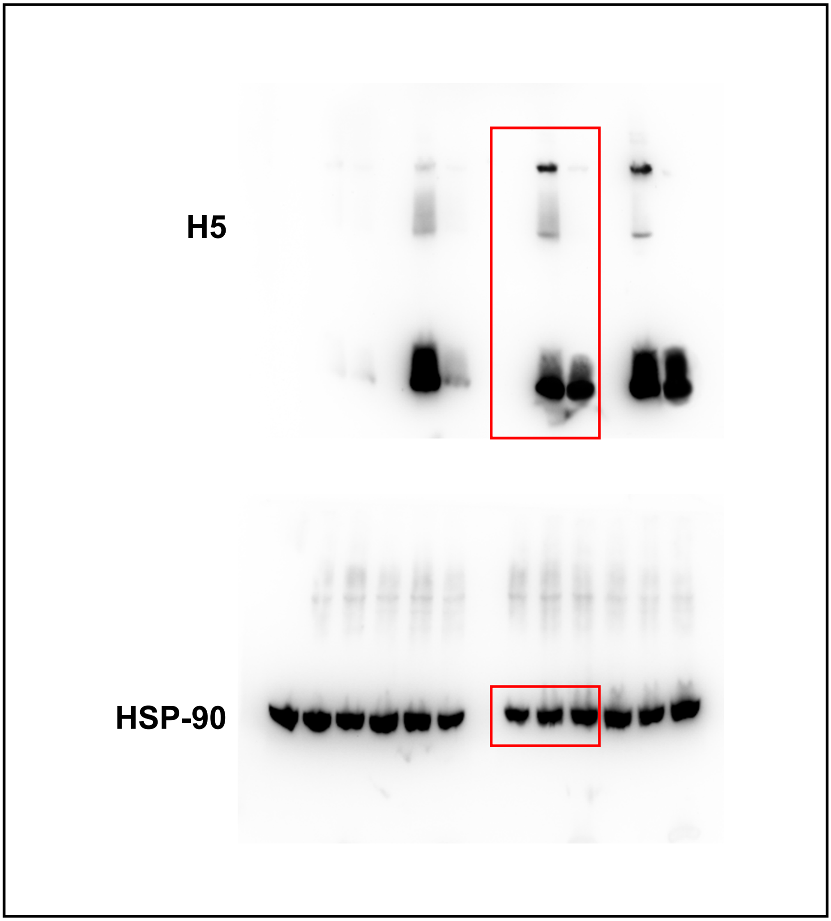

Fig 7E

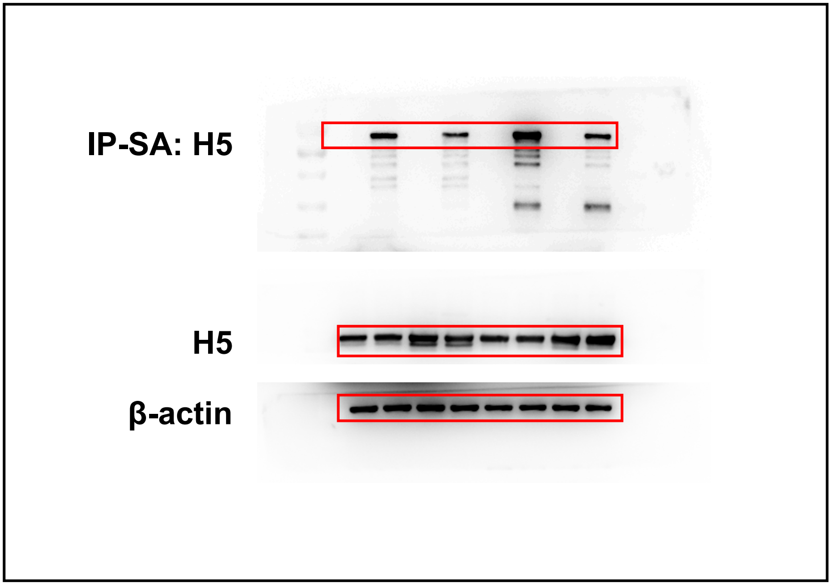

Fig 7F

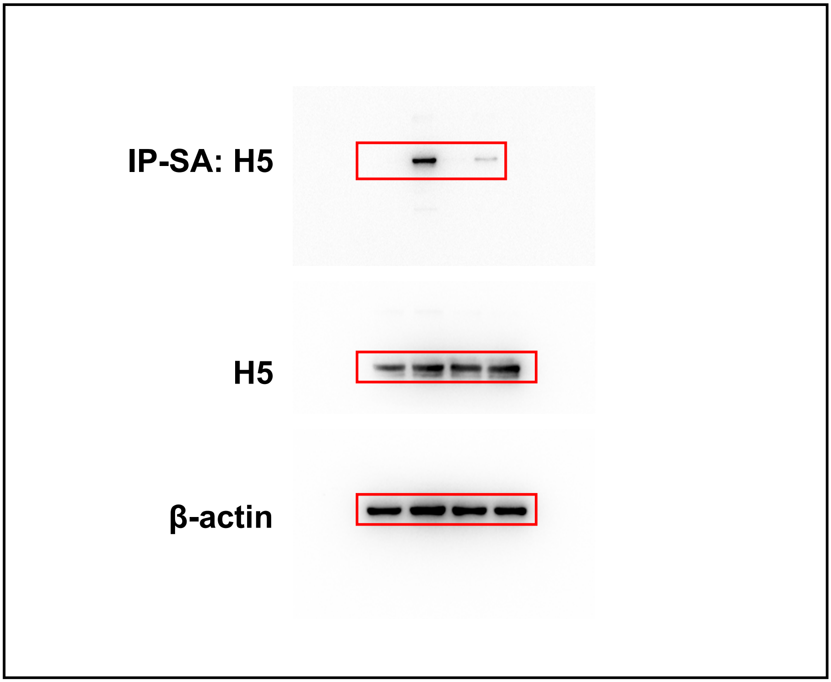

Fig 7K

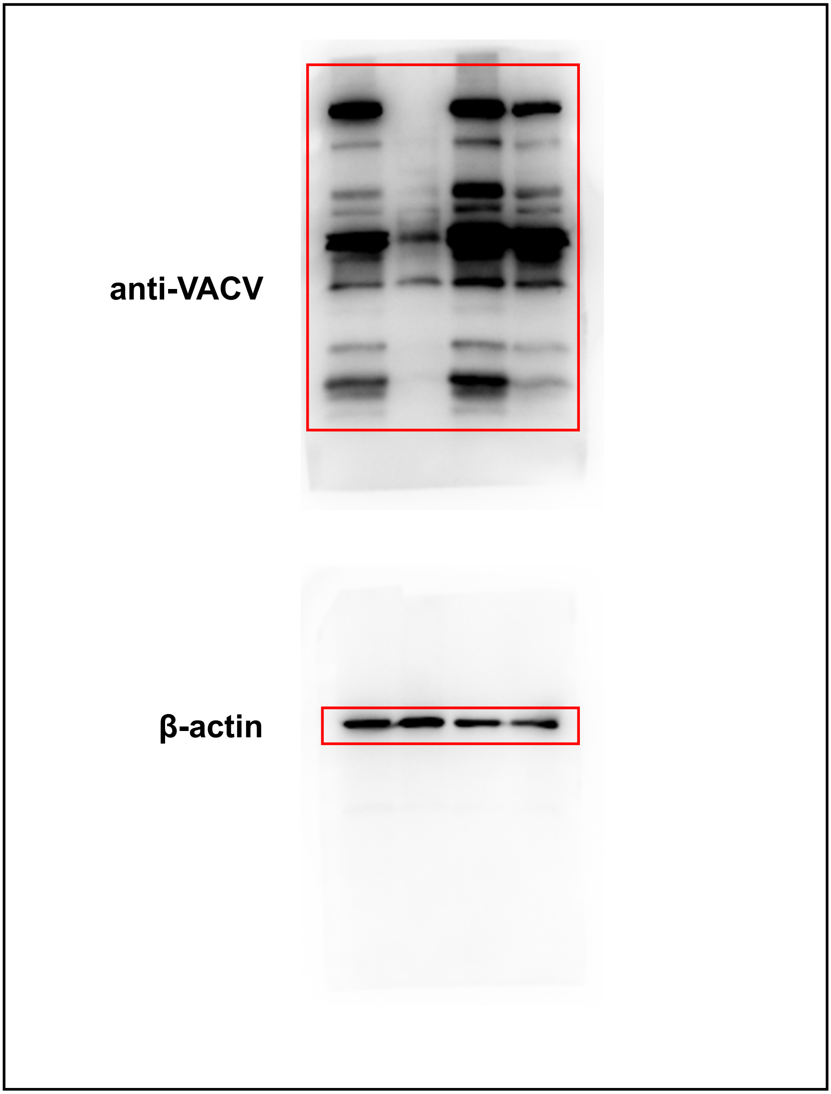

Fig 7N

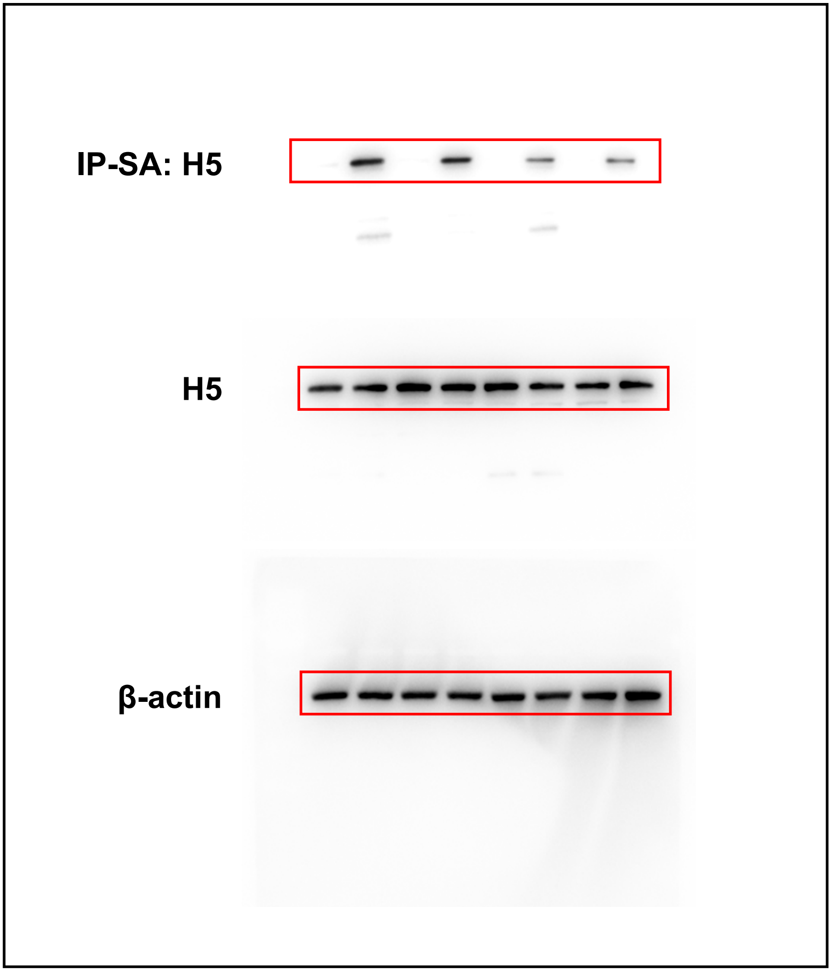

Fig S1A

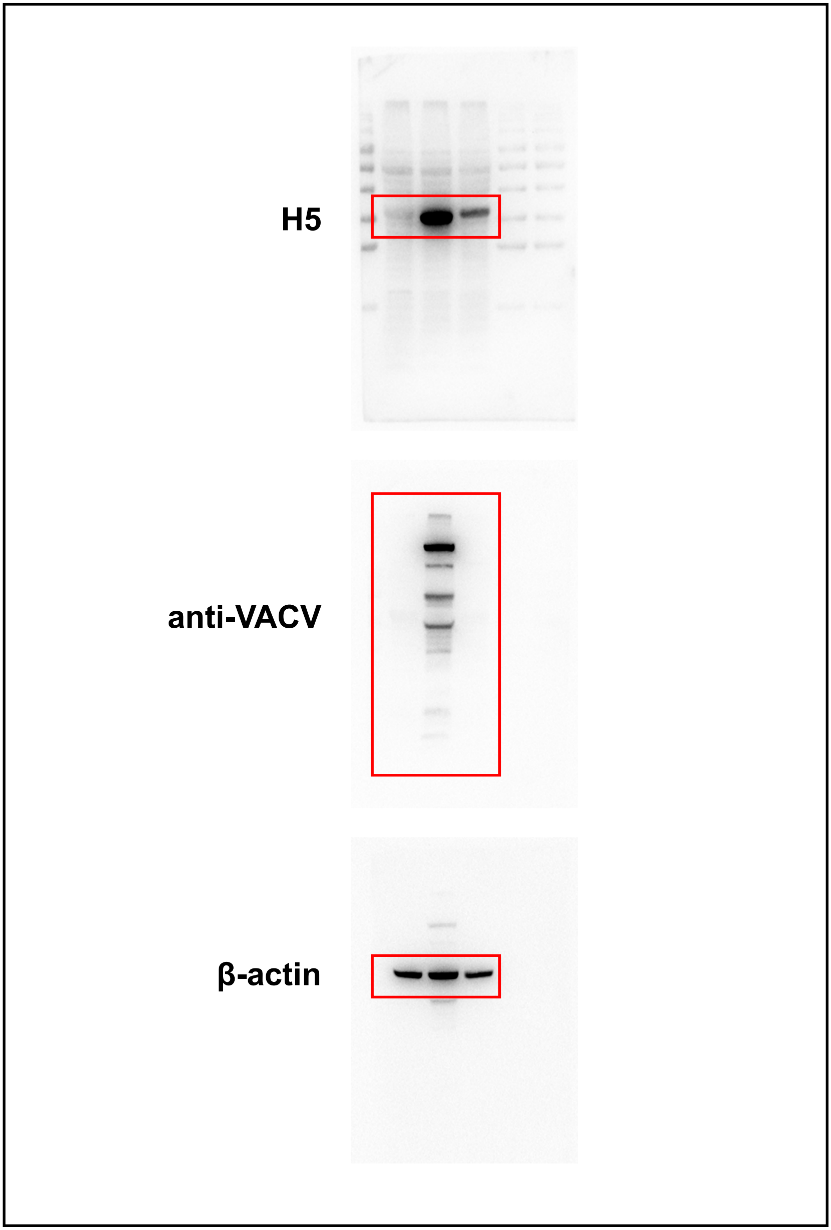

Fig S6

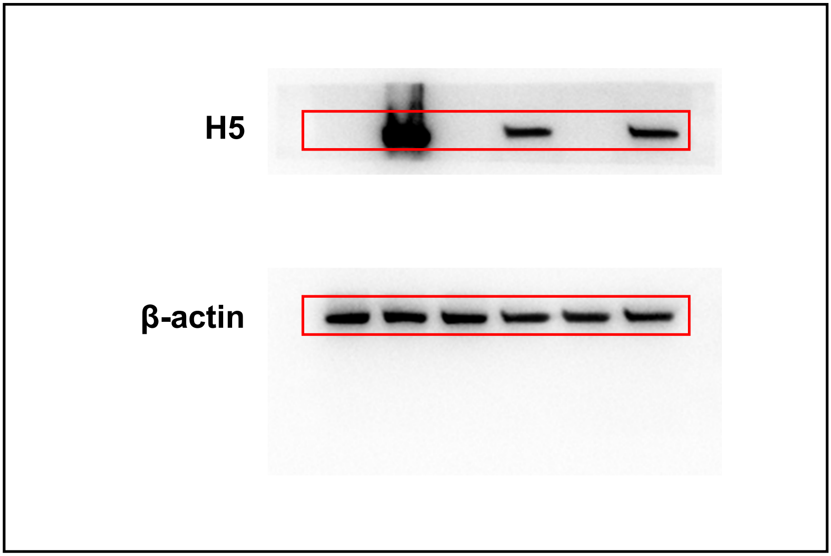

Fig 1C

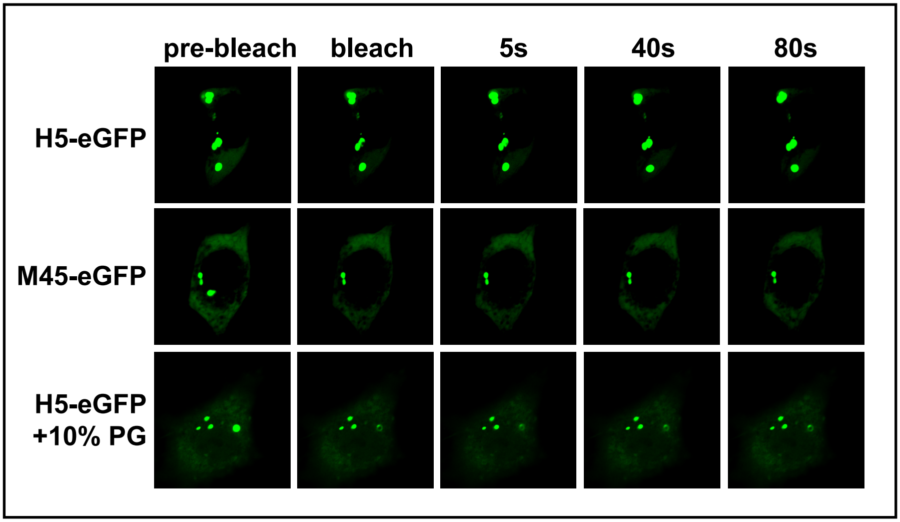

Fig 1E

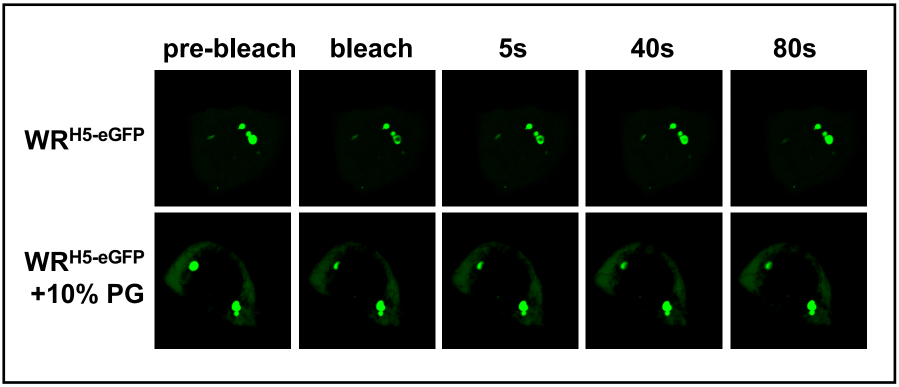

Fig 1G

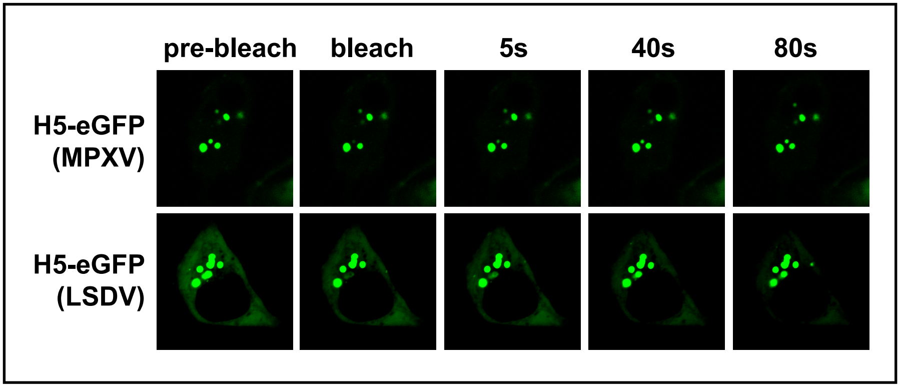

Fig 2D

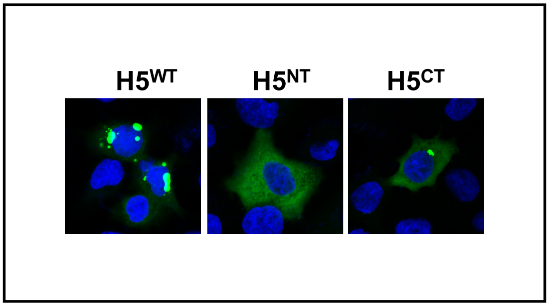

Fig 2E

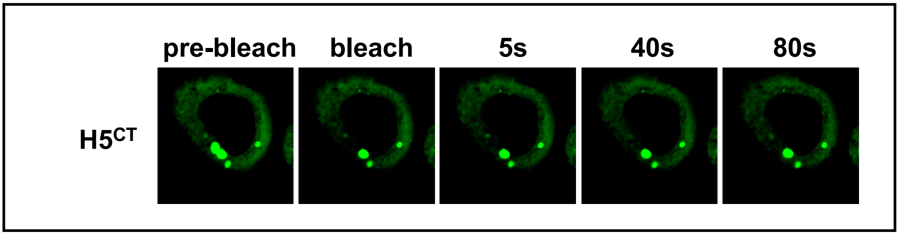

Fig 3B

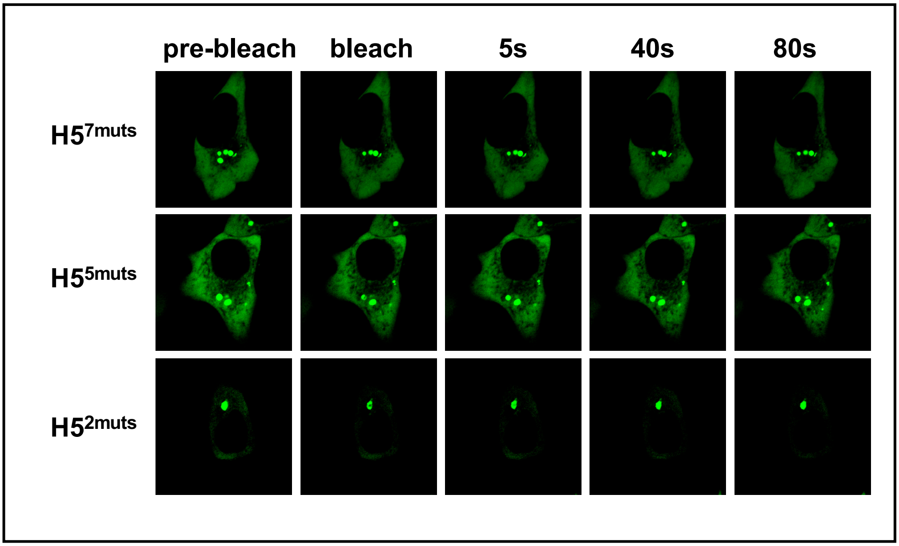

Fig 3E

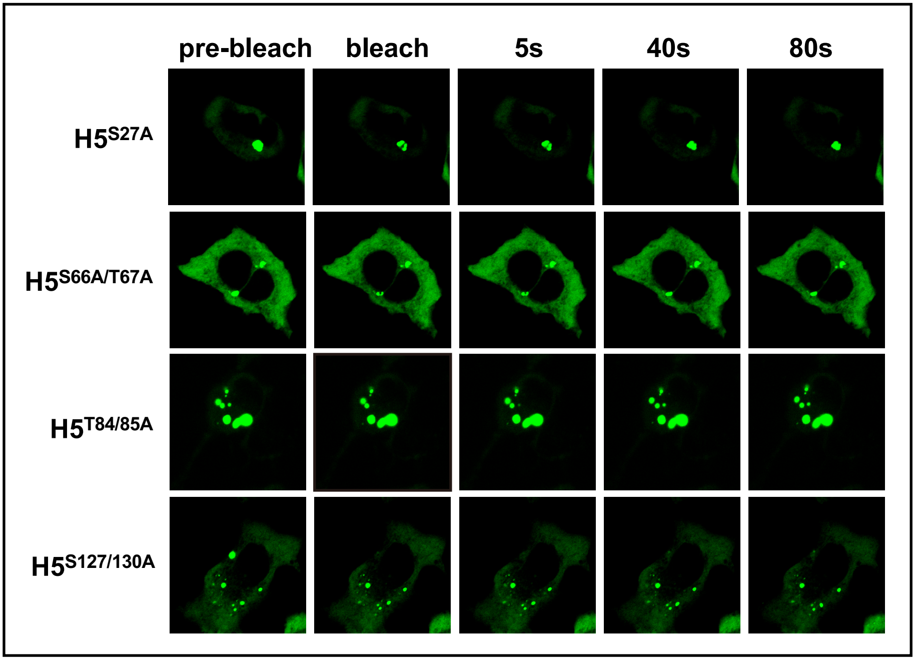
pre-bleach
bleach
5s
40s
80s
H5S27A
H5S66A/T67A
H5T84/85A
H5S127/130A

Fig 4B

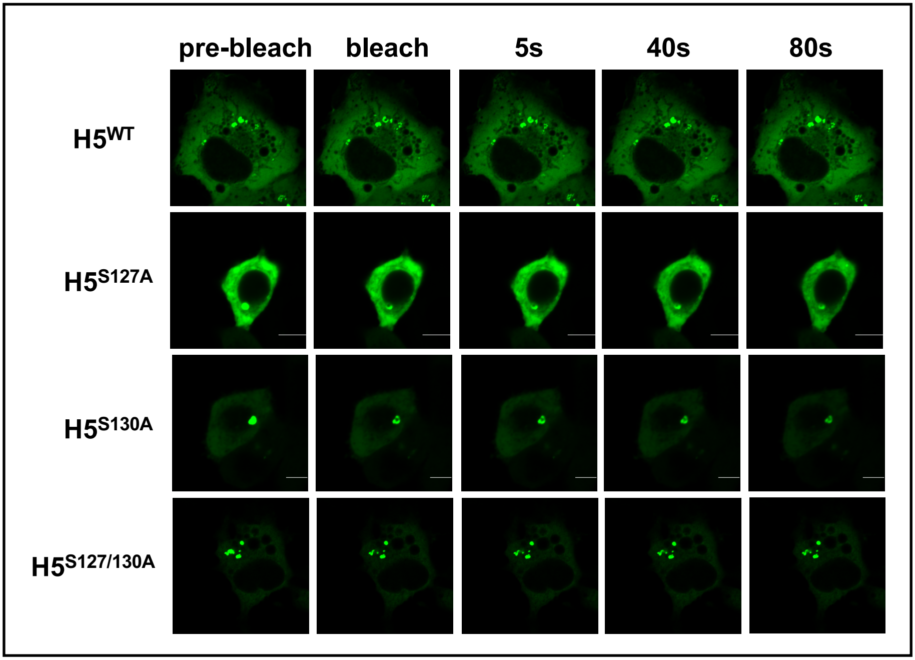
pre-bleach
bleach
5s
40s
80s
H5WT
H5S127A
H5S130A
H5S127/130A

Fig 5A

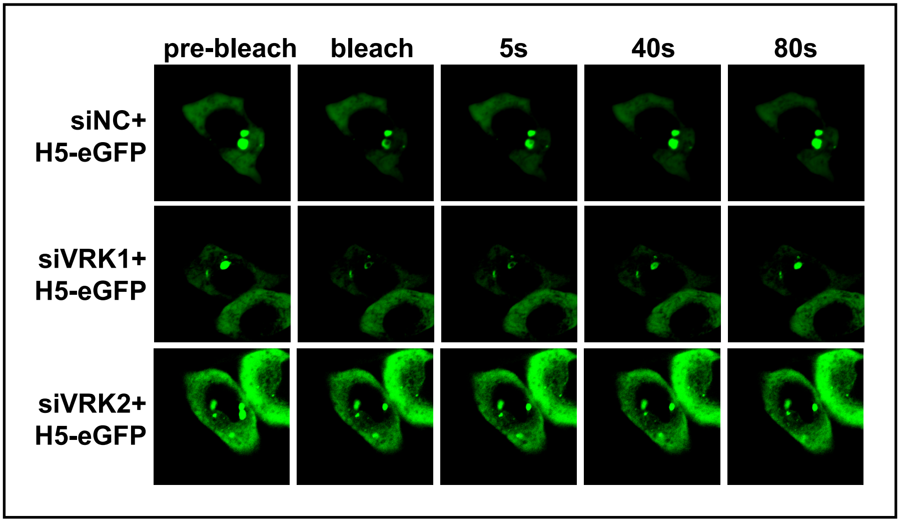
pre-bleach
bleach
5s
40s
80s
siNC+
H5-eGFP
siVRK1+
H5-eGFP
siVRK2+
H5-eGFP

Fig 5C

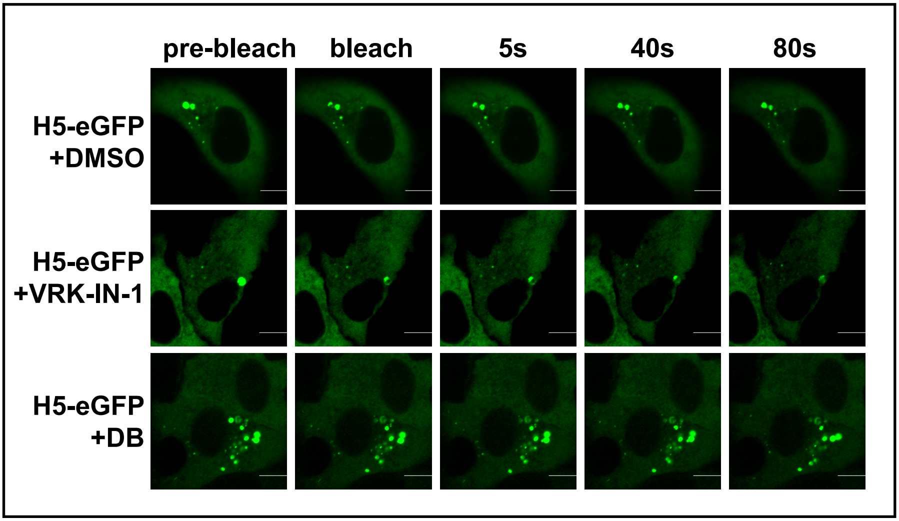
pre-bleach
bleach
5s
40s
80s
H5-eGFP
+DMSO
H5-eGFP
+VRK-IN-1
H5-eGFP
+DB

Fig 5E

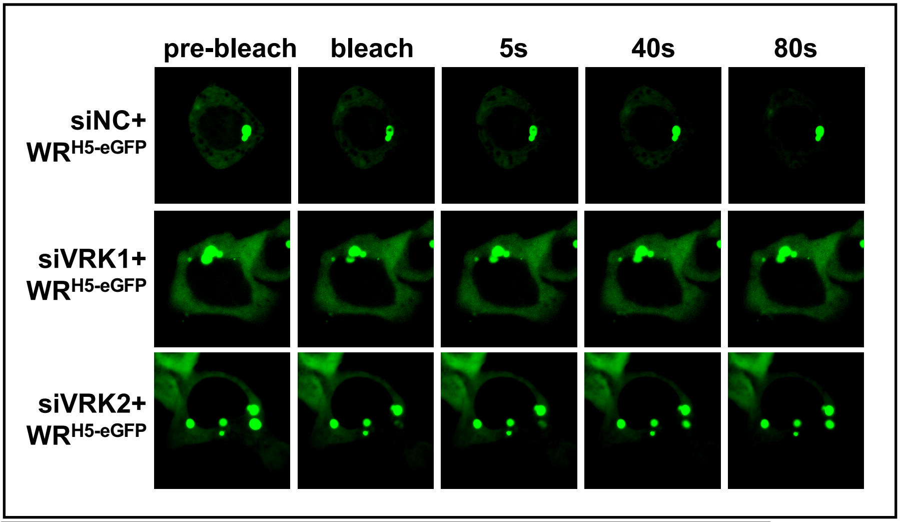

Fig 6B

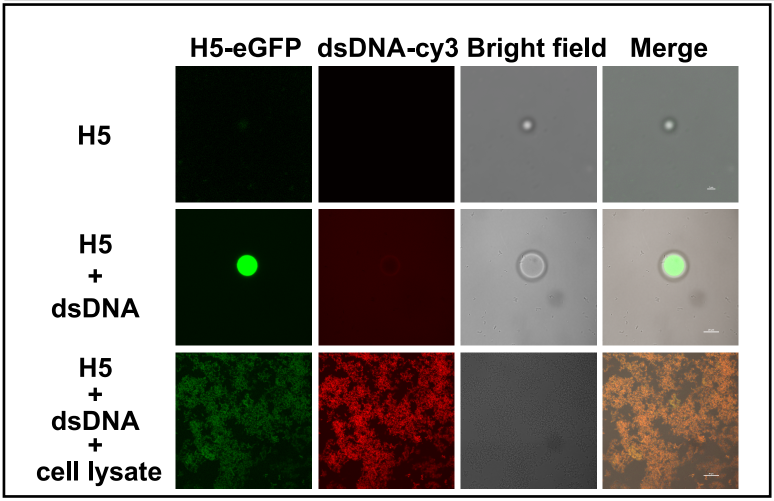

Fig 6C

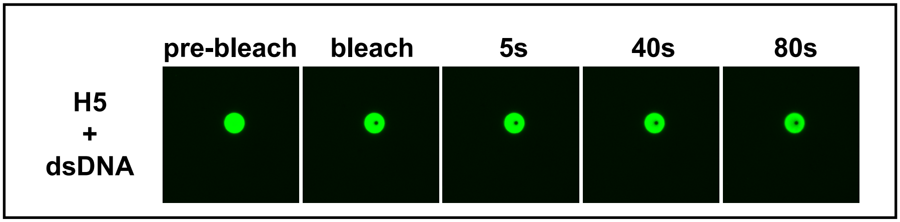

Fig 6E

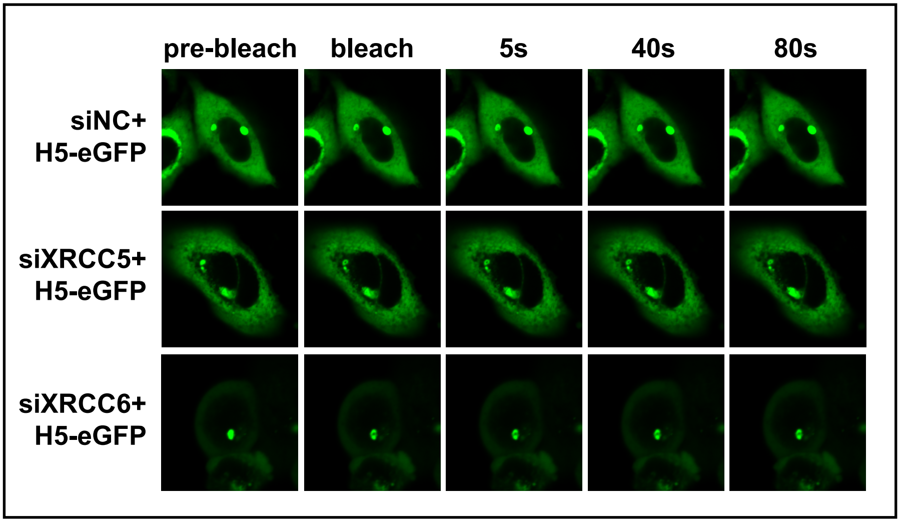

Fig 6G

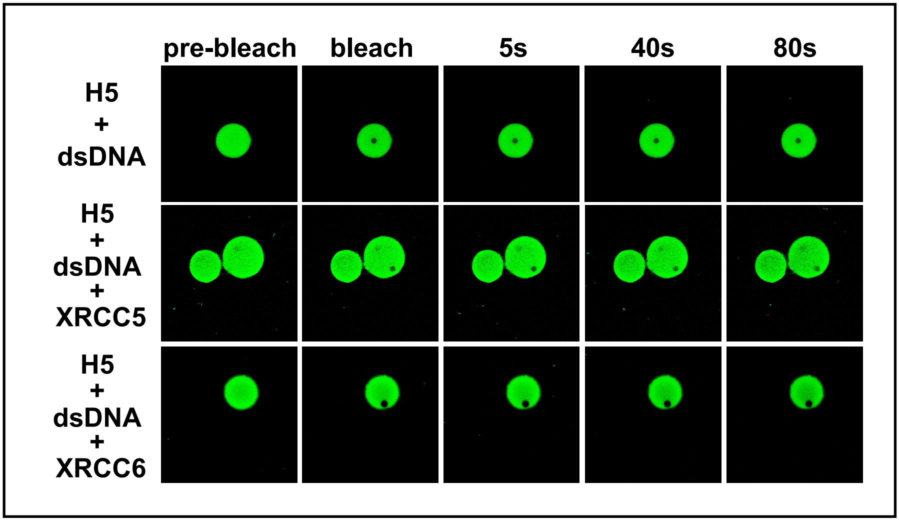

Fig 7G

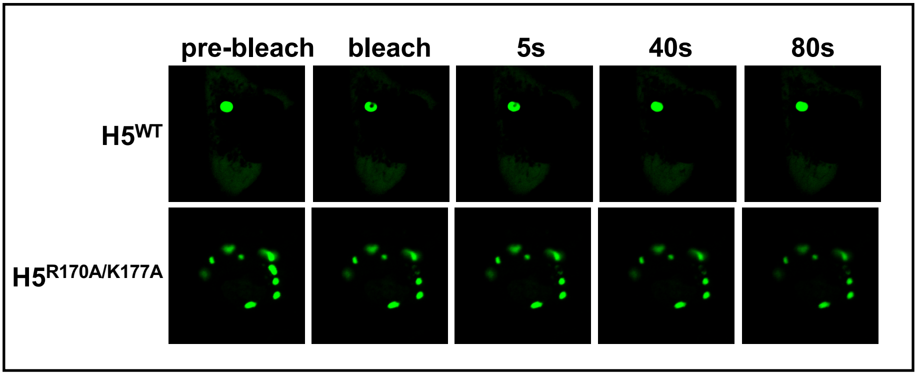

Fig 8A

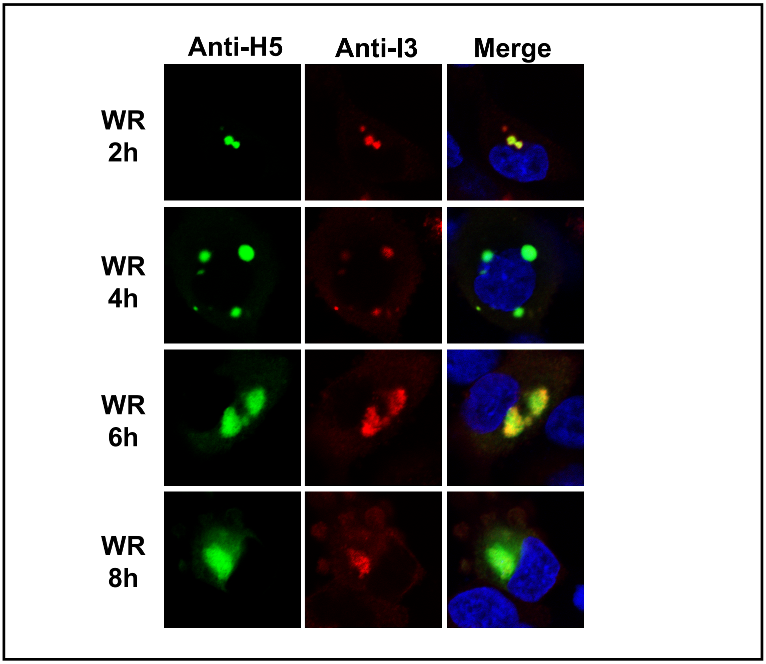

Anti-H5
Anti-I3
Merge
WR 2h
WR 4h
WR 6h
WR 8h

Fig 8B

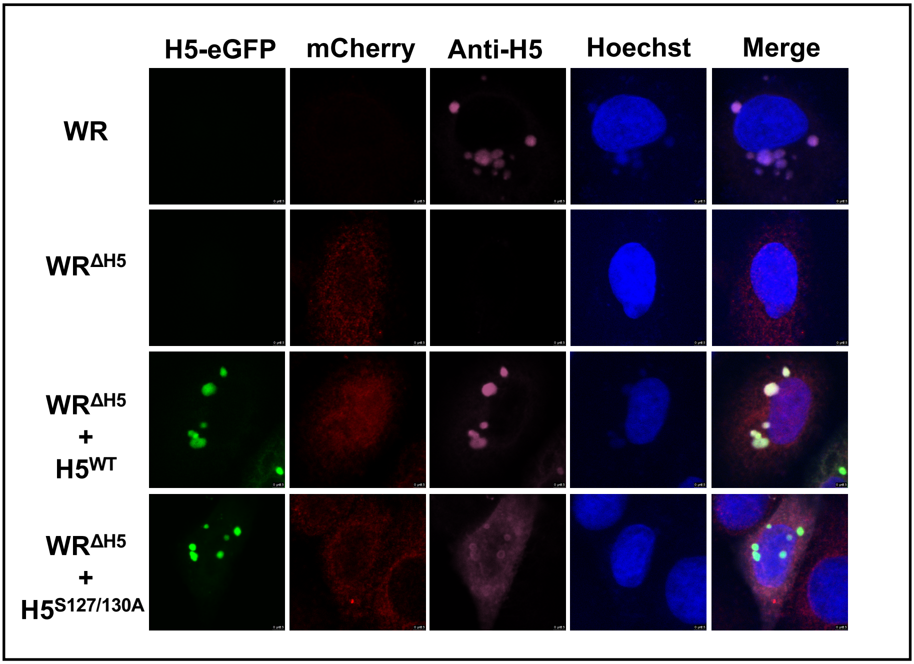

H5-eGFP
mCherry
Anti-H5
Hoechst
Merge
WR
WRΔH5
WRΔH5 + H5WT
WRΔH5 + H5S127/130A

Fig 8E

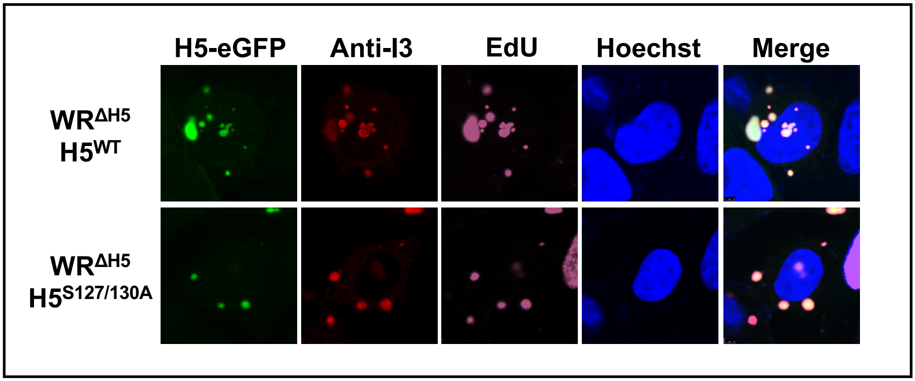

Fig S1B

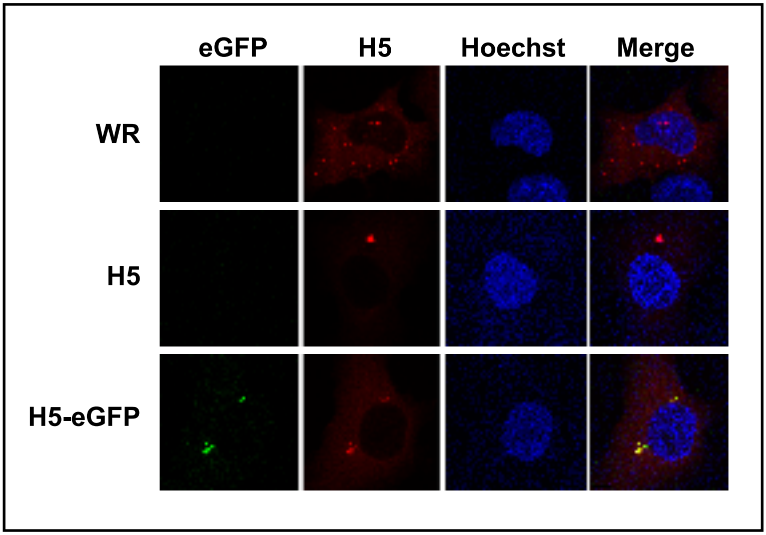

Fig S5D

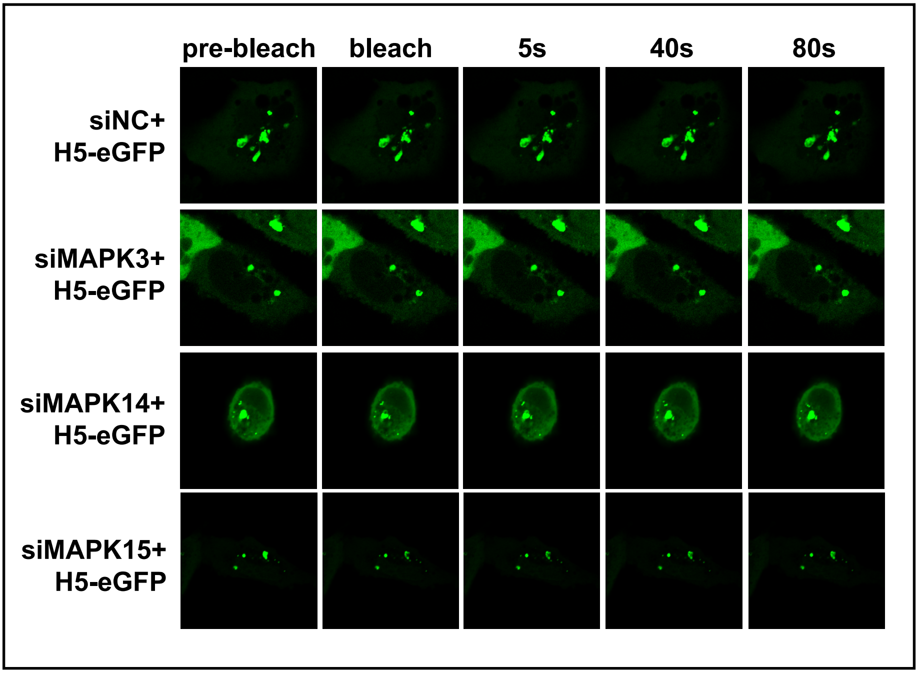
pre-bleach
bleach
5s
40s
80s
siNC+
H5-eGFP
siMAPK3+
H5-eGFP
siMAPK14+
H5-eGFP
siMAPK15+
H5-eGFP

Fig S7

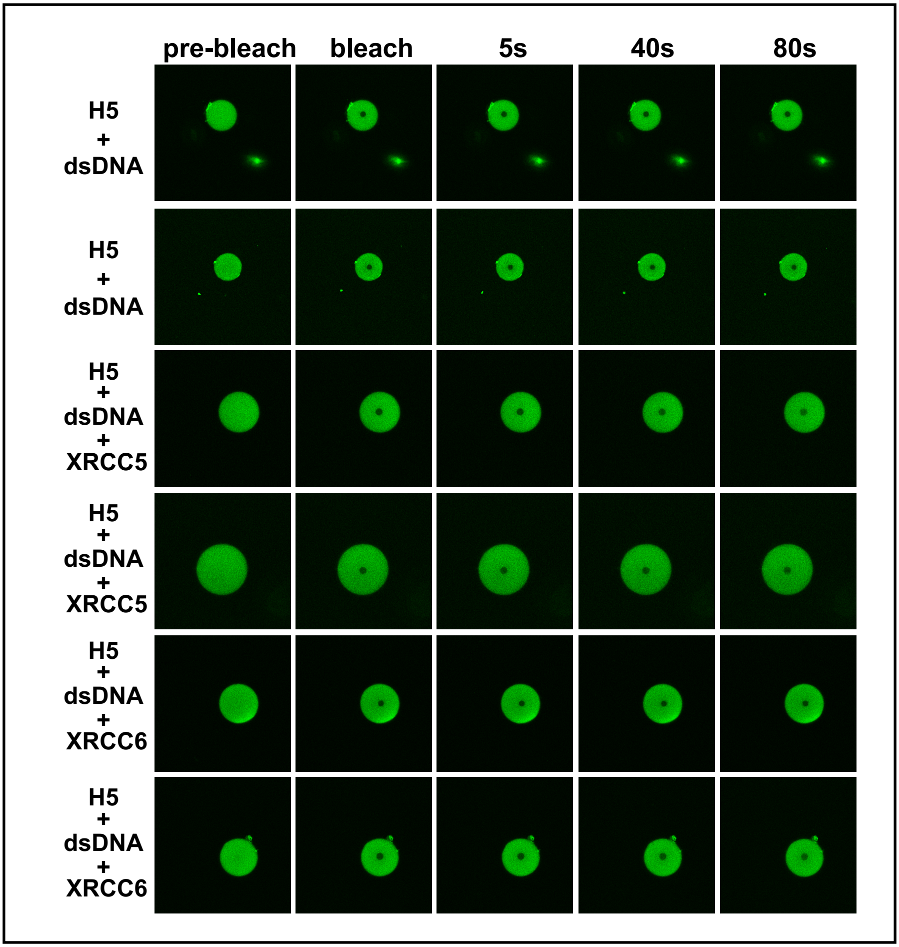

pre-bleach
bleach
5s
40s
80s
H5 + dsDNA
H5 + dsDNA
H5 + dsDNA + XRCC5
H5 + dsDNA + XRCC5
H5 + dsDNA + XRCC6
H5 + dsDNA + XRCC6
